# Measuring time use in rural India: Design and validation of a low-cost survey module

**DOI:** 10.1016/j.jdeveco.2023.103105

**Published:** 2023-09

**Authors:** Erica Field, Rohini Pande, Natalia Rigol, Simone Schaner, Elena Stacy, Charity Troyer Moore

**Affiliations:** a Duke University, United States of America; b Yale University, United States of America; c Harvard University, United States of America; d University of Southern California, United States of America; e University of California, Berkeley, United States of America

**Keywords:** Time use, Labor supply, Gender, Field experiment, Measurement, Validation

## Abstract

Time use data facilitate understanding of labor supply, especially for women who often undertake unpaid care and home production. Although assisted diary-based time use surveys are suitable for low-literacy populations, they are costly and rarely used. We create a low-cost, scalable alternative that captures contextually-determined broad time categories; here, allocations across market work, household labor, and leisure. Using fewer categories and larger time intervals takes 33% less time than traditional modules. Field experiments show the module measures average time across the broader categories as well as the traditional approach, particularly for our target female population. The module can also capture multitasking for a specific category of interest. Its shortcomings are short duration activity capture and the need for careful category selection. The module’s brevity and low cost make it a viable method to use in household and labor force surveys, facilitating tracking of work and leisure patterns as economies develop.

## Highlights

• We use field experiments to assess how well a new survey module captures time use and compares to a more traditional approach.
• It accurately captures average time use, and is lower-cost, shorter, and easier to administer than a widely-used approach.
• Module weaknesses are capture of short duration activities and need for careful category selection.
• It was developed for low-literacy, rural women and assessed trade-offs of interest for them, but it can readily be adapted.
• The module’s brevity and low cost make it a viable approach to measure time use in regular household and labor surveys.

## 1 Introduction

Understanding how people spend their time can provide critical insight into how individual behavior and gender roles change over the course of economic development.^1^ However, because reliable time use data are notoriously difficult and expensive to collect, they are rarely available in nationally representative surveys, particularly in low-income settings (Hirway, 2010). In our focus country, India, no large-scale time use data were collected from 1998 to 2019, a period during which female labor force participation fell from 31% to 21% (with declines concentrated in rural areas) (World Bank Group, 2021). Standard household surveys document this decline alongside large gains in women’s education, but in the absence of time use data, they cannot determine whether women reallocated time to leisure, unpaid work (within or outside the home) or childcare, choices with very different implications for household dynamics and welfare.

Time use data in low-income rural communities, where men and women’s labor supply can change dramatically during processes of economic development, is invaluable for policy but particularly difficult to collect. Data must be collected with care, taking into account cultural context, a higher incidence of passive caregiving, and multitasking, while avoiding classification errors when households perform the same activities for productive and consumptive purposes (Charmes, 2006, Seymour et al., 2020, Hirway, 2022). Lower literacy rates make self-administered time diaries challenging, and low digital literacy limits use of smartphone-based solutions that circumvent reading and writing barriers.^2^ The current status quo of enumerator-administered diary modules requires a significant amount of survey time and enumerator expertise. This makes regular collection of time use data at scale difficult and costly, as well as subject to high rates of attrition and measurement error (Buvinic and King, 2018).

In this paper, we propose and validate a “stylized diary hybrid”, or “Hybrid” approach for collecting time use data in a short period at a low cost. We demonstrate that this method meets three important criteria: first, it is appropriate for a less literate population; second, it is shorter to train and administer – and, therefore, less costly – than an enumerator-assisted diary approach; third, it is equally accurate as a traditional assisted diary in assessing average time use for the targeted set of activities and population for which it is designed. We conclude that this module offers a promising time use data collection method that can easily be scaled to fit into, say, a national household survey conducted by a statistical institute in a lower-income country.

The method is a “hybrid” of the assisted time diary approach, in which an enumerator assists the respondent in filling out a time diary by reconstructing a reference day, and stylized survey questions that collect self-reported time spent in broad activity categories. Respondents relay time use for the previous day to the enumerator, who allocates one token per hour across cards with photos depicting the activity category. After narrating their day, respondents review and refine token allocation with enumerators. The specific module we consider uses eight activity categories chosen with the goal of distinguishing between time spent on paid work, unpaid work, and leisure for rural Indian women. Following qualitative scoping with the study population of largely agricultural, low-income households, we selected these eight context-specific groupings — wage work, self-employment, working on one’s own field, chores outside the household, chores inside the household, sleeping, leisure and caring for family members.

We validate the Hybrid module among low-income rural Indian households by comparing data quality and module performance to two other methods: the survey-based assisted diary method used by India’s National Sample Survey Office to collect time use data (henceforth the “Traditional” approach) and a resource-intensive “Gold Standard” method. The Gold Standard employs short, high-frequency visits to respondents’ homes to record day-of activities with the least amount of recall error possible. We randomize the method deployed across subjects and within subjects over multiple visits in our validation exercise.

Our experimental results show that the Hybrid module captures average time use by category well. Differences from the Gold Standard are typically low in magnitude, and within-person comparisons show that the method is no less accurate than the Traditional approach at capturing category-wise time use. When we assess the module’s performance by gender and life stage, the Hybrid continues to perform well relative to both the Gold Standard and Traditional approaches among women, the population for which it was originally developed. In contrast, it tends to overestimate unmarried men’s leisure time at the expense of time spent on chores. Our hypothesis is that the relatively worse performance among unmarried males likely reflects priming or desirability bias, since photographs depicting time spent on household chores featured a focal woman, rather than a man.

In terms of both field time and enumerator training requirements, the Hybrid module is substantially less expensive to use. For a large sample survey, we estimate that the Hybrid approach yields cost savings of about 25% over the Traditional module. Furthermore, the Hybrid module uses fewer time use categories and larger time intervals, resulting in a 33% (five minutes) faster completion time than the Traditional module, which has the potential to improve survey compliance and data quality. According to our enumerator team, who administered the module in the context of a long household survey prior to the validation study, the brevity and ease of administration reduces enumerator and respondent fatigue. Finally, the module’s simplicity makes it appealing for respondents in low-education settings, especially women for whom it was designed. Enumerators reported that our respondent population easily understood and engaged with the module.

The Hybrid module, by design, covers a limited set of activities and lacks detail when compared to typical time diary data. The majority of activities, for example, are collected in hours rather than minutes, as required by the use of tokens. And while the module captures average time use well, our analysis suggests that it performs worse than the Traditional module when it comes to recording low duration activities. The structure of the module also limits its ability to capture multitasking in its entirety. Using passive caregiving as an example, we show how this shortcoming can be addressed for specific short duration activities that are prone to multitasking but are of interest to researchers.

Category selection is a critical design choice for the Hybrid module, as appropriate categories depend on research questions and local context. Thus, we encourage researchers using the Hybrid approach to use qualitative fieldwork to inform their choice of time use categories and wording and photos used to describe them, as well as to consider categories that can distinguish market and non-market work as it relates to their population of interest. This pre-work is particularly important to ensure photographs are tailored to the population of interest and avoid priming based on gender-stereotypical time allocation.

In addition to creating a high-performing, simple-to-implement time use module that targets low-income groups, our work makes a methodological contribution. It has been challenging to quantify the advantages and disadvantages of various approaches to gathering time use data since different techniques are rarely used across statistically equivalent populations (Kan and Pudney, 2008).^3^ We validate the Hybrid module by randomizing the method used to collect time use data from respondents during repeated visits. This allows us to rigorously assess the relative accuracy of different methods of time use data collection conducted on the same population and to experimentally test for priming effects. It also allows us to build an accurate estimate of the relative costs of different methods.

The remainder of the paper proceeds as follows: Section 2 reviews time use data collection techniques, the Hybrid module and time use methods to which we compare it. Section 3 describes our study population and validation experiments. Section 4 presents our main results, discussing the module’s overall performance in terms of data quality and ease and cost of implementation. Section 5 concludes.

## 2 Background: Time use data collection

### 2.1 Existing approaches to time use data collection

Collecting individual time use data is difficult and expensive. The most common techniques for gathering data on time use are shown in Table 1. Self-administered diaries, in which the respondent fills a time diary either in real time or retrospectively, are often used in higher income settings because they do not require enumerator time, yield rich data, and can account for multitasking. For respondents with low literacy, such methods are often infeasible, although recent initiatives like the graphical smartphone-based self-administered diaries by Daum et al. (2019) show promise.^4^

An alternative to self-administered diaries is an “assisted” diary approach delivered directly by an enumerator. Respondents narrate their activities in chronological order, and enumerators ask pre-determined probing questions to assess and categorize the activity, while occasionally collecting additional contextual data, such as where the activity occurred. Although this technique is sometimes employed in low-income countries (such as India), it is time-consuming, cognitively demanding, requires competent surveyors, and has nontrivial training costs (Seymour et al., 2020, Hirway, 2022).

**Table 1:** Commonly used approaches to collect time use data.

Method	How administered	Frequently used in	Advantages	Disadvantages	Survey examples
Time diaries (Self-administered)	– Respondents themselves fill diary either in real-time or retrospectively	– Stand-alone national time use surveys – Higher-income/education populations	– Can gather very detailed and comprehensive information – Can account for simultaneous activities – Can reduce measurement error since reported hours must add up to 24	– Detailed versions are time-intensive – Can require significant training depending on respondent population	– American Time Use Surveys – Eurostat Time Use Surveys – Pictorial smartphone-based time diary developed for low-literacy populations (Daum et al., 2019)

Time diaries (Enumerator- administered)	– Respondent retrospectively reports to enumerator on activities in chronological order for specified time period	– Stand-alone national time use surveys – Modules in longer household surveys	– Can gather very detailed and comprehensive information – Can account for simultaneous activities – Can reduce measurement error since reported hours must add up to 24 – Can rely on trained enumerator for more consistent reporting	– Detailed versions are time-intensive, can result in fatigue – Can require significant enumerator training – May be prone to social desirability bias	– Indian Time Use Survey: – 1998 (pilot in 6 states) – 2019 national survey

Stylized questionnaire	– Respondent aggregates and reports on time involved in specific activities over set period (e.g., one week) as part of enumerator-administered survey	– Module within national household surveys – Low-income settings (e.g., used in women’s empowerment in agriculture index)	– Easier to administer to populations with less sense of time – Can be tailored to specific types of time use – Can fit into larger household survey – Shorter and lower-cost than other approaches	– Cognitive burden can increase time needed to administer – Recall bias; telescoping, social desirability bias, may affect responses – Does not account for simultaneous activities or time of day/chronological order – May over- or under-count time that should add up (e.g., 24 hours of the day)	– Argentina 2001 Survey of Living Conditions – 2005 Bangladesh Household Income and Expenditure Survey – 1998–99 Nicaragua Living Standards Measurement Survey – 2002 Mexican Family Life Survey – 2016 Young Lives Survey

Experiential sampling methods	– Respondents contacted at random intervals and asked to report their activity in real-time, often alongside reporting on perceived well-being or emotional state	– Behavioral surveys – More commonly done with high-income populations	– Avoids retrospective reporting biases – Can gather measures of subjective well-being alongside time use – Can cover relatively longer time periods than most approaches – Nature of short responses can be less burdensome	– Systematic non-response (by individuals or activities) – Tends to focus on specific episodes rather than paint full picture of time use, or else is time-consuming and may generate respondent fatigue	– German Socio-Economic Panel – Tested in Kenya with low-income rural population (Cook et al., 2022)

Observation-based	– Enumerator shadowing or observation	– Used infrequently due to cost and complexity	– Avoids retrospective reporting biases – Can be used in populations with less sense of time and low literacy	– Potential for Hawthorne effects – Costly per person implementation – Costly and time-consuming enumerator training	– Bangladesh Bureau of Economic Research Survey of Intra-Household Distribution and Poverty Incidence (2004)

Authors’ synthesis utilizing the following source documents: (Alkire et al., 2013, Anusic et al., 2017, Charmes, 2015, Chenu and Lesnard, 2006, Cook et al., 2022, Daum et al., 2019, Hirway, 2022, Jagnani, 2022, Khondker, 2006, Masuda et al., 2014, Seymour et al., 2020, National Research Council, 2000, United Nations Development Programme, 2018).

Since diary-based collection is often infeasible for large samples in lower-income countries, time use data collection in these settings has frequently relied on stylized survey questions that ask individuals to aggregate time over a given reference period (e.g., “How much time did you spend cooking yesterday?”). According to research, this produces higher measurement error when compared to detailed diaries, and errors vary systematically by respondent factors like gender or number of working hours (Kan and Pudney, 2008). Higher error rates for female respondents may reflect the non-contiguous, multi-tasking nature of household work and home-based production. Behavioral factors such as telescoping, availability heuristics, and social desirability bias are also likely to affect stylized questions (Kahneman et al., 2004), and this approach is often more cognitively burdensome for respondents than appreciated (Seymour et al., 2020).

As seen in Kahneman et al. (2004) and Cook et al. (2022), experiential sampling methods have been used to combine data on time use with subjective measures of welfare. While valuable in their own right, experiential methods typically evaluate activities and well-being at random intervals, and are therefore less well-suited to gathering complete data on time use.

A final possibility is observation-based time use data collection, which requires enumerators to monitor individual respondents throughout the course of an entire day. This method aims to build a comprehensive picture of a respondent’s activities through a specified interval. Although errors in respondent recall and misreporting are addressed by this strategy, it is significantly more expensive and might be subject to Hawthorne effects. For these reasons, this technique is rarely applied.

According to Hirway (2010), only a few countries have gathered national time use data more than once, and about half of reported time use data collection operations in low and middle-income nations were only pilots or small-scale surveys. Furthermore, little research has been done to quantify measurement error or evaluate the relative accuracy of the various techniques of time use data collection. A recent review of time use data across the globe highlights how important it is, particularly for developing countries, to integrate time use survey modules into ongoing data collection in order to better understand links between time, employment, and respondent demographics, as well as the evolution of household and market activities (Buvinic and King, 2018). Our method aims to address this need, and fill gaps in the evidence base for important policy decisions related to gender, employment, caregiving, and safety net programs (Data2x, 2018).

### 2.2 Time use data collection: Our validation approach

Our validation exercise compares three methods for collecting time use data: two survey-based – the Traditional and Hybrid methods – and one observation-based, “Gold Standard” method. While the Traditional Assisted Diary approach was adapted directly from survey protocols used for at-scale survey data collection by the government of India, both the Gold Standard and Hybrid method were developed on the basis of qualitative work. We provide more details on each below.

#### Traditional assisted diary.

This method was based on the 1998/99 Indian Time Use Survey conducted by India’s Ministry of Statistics and Planning.^5^ A respondent was interviewed about her activities, in chronological order, during the previous day. She could report up to six separate activities completed within any given hour, with activity duration captured in minute increments. Enumerators also recorded whether the respondent performed passive childcare during each 15 min interval, as well as the location (inside/outside the household) and nature (paid/unpaid) of each activity. Enumerators classified time use using the 152 Indian Time Use Survey categories.

#### Gold standard method.

This method used a modified version of in-person observation of actual time allocation, which consisted of multiple brief interviews on the day of the visit. We first piloted a traditional observation-based approach, in which enumerators stayed in households and unobtrusively observed respondent activities for a fixed time. That pilot suggested standard observation methods were too intrusive for our study population, as women interrupted daily activities to interact with the enumerator. We also trialled an approach in which we gave respondents mobile phones and called them throughout the day to collect time use data. This did not work well; given our respondents’ limited literacy and comfort with mobile phones, some refused to participate, and others often did not answer the phone as they forgot to carry it with them.

Our final protocol was designed to collect real-time data throughout the day without altering respondent activities. During the reference day, the enumerator visited the respondent every hour within a 10-hour window. After the initial visit, each was 2-3 min. The respondent was asked what she had done since the enumerator last visited. Activities, coded using the same 152 categories as above, were coded in minutes, and up to six activities could be recorded in a given hour, with passive care available as a cross-cutting simultaneous activity, as in the Traditional method. Each activity’s location (inside/outside the household) and nature (paid/unpaid, along with method of payment) were also recorded.

To ensure that respondents did not adapt activities due to Hawthorne effects or because they anticipated additional visits, our survey protocols and scripts, available in the Appendix^6^, instructed respondents to go about their day as usual, regardless of activity location, throughout the day. For those who left home during the day, we reconstructed time use for the entire day by collecting information about activities upon return.^7^ No observation visits occurred between the hours of 6 pm and 6 am. Instead, on the day’s final visit, enumerators obtained the respondent’s planned rest-of-day activities and associated timing. They also obtained retrospective data on this time period the next day. As prospective and retrospective reports were nearly identical, we use the prospective reports.

Our Gold Standard approach aimed to reduce measurement error due to recall while limiting disruption in households’ schedules and minimizing the possibility that participants altered activities due to a stranger in the household, giving us as close a measure as possible to their natural time allocation. Data on 236 unique days where enumerators logged visits using GPS show they visited households an average of 8.4 times during the Gold Standard day (with a median and mode of 9 visits). The average and median time between Gold Standard visits was 60 min, with the 10th and 90th percentile of time elapsed at 55 and 63 min, respectively. In short, protocols were closely followed.

#### Stylized diary hybrid.

The Hybrid method was developed in the context of a multi-topic endline survey described in Field et al. (2021). We needed a method to quickly collect time use data that required minimal explanation (given the low rates of literacy in our population) and would minimize fatigue in an already lengthy survey. Our protocol builds on the participatory rural appraisal (PRA)-inspired method used in Masuda et al. (2014), in which respondents allocated pieces of macaroni representing 20-minute intervals across 15 activity cards.^8^

To map individual activities into contextually relevant time use groupings, we began by conducting open-ended, semi-structured conversations with women in which we asked respondents to explain what they had done during the previous day. Our objectives were to identify: (i) major time use categories relevant to our population, and (ii) categories that can quantify the extent to which women engage in paid and unpaid work, with a focus on how much production occurred inside versus outside the home (to better understand women’s mobility, which is limited in this part of India). Key focus areas included *where* respondents reported undertaking activities and *why* activities were undertaken — for income generation, consumption, or both.

As one example of how this work informed category construction, we discovered that women underreported income generating activities, describing tasks like livestock care purely as household chores, despite the fact that the household occasionally sold outputs like milk or eggs. We, therefore, separated time use categories for pure home production versus home-based work that generated income. Separating indoor household chores (which rarely had an income generating objective) from outdoor household chores (where activities were more likely to include household and market objectives) focused the categories and made it easier to distinguish between market and non-market work.

Our formative research also highlighted the risk of underestimating one important repeated activity for women — caring for children while undertaking other activities. When asked about their time use, women often did not report or even recognize that they had engaged in “passive” childcare. A woman may, for example, care for a child while preparing a meal, feeding livestock, or gathering water, but she may not consider this worth reporting (or she may not recognize it herself) because it is not her primary objective. Reflecting our research aims, the qualitative work and our time use categories were “centered” on women, with the goal of distinguishing key activities in women’s daily lives to shed light on her economic engagement and autonomy. Had we focused our qualitative research on another group, say young men, our categories might have differed, emphasizing activities like market work, job search, and human capital accumulation.

We also conducted an intensive “pre-pilot” with 12 respondents in which we captured time use for full days using the 152 Indian Time Use activity categories to see how well women’s detailed time use mapped onto our set of potential time use categories.^9^

Ultimately, we chose the following eight categories based on our qualitative and pre-pilot findings: wage work, self-employment, working on one’s own field, chores outside the household, chores inside the household, sleeping, leisure and caring for family members. These activities were easily represented using a set of context-specific photos, which helped respondents anchor categories and engage with the exercise.^10^

Our implementation approach differed from existing PRA-style work in that enumerators performed the initial time allocation activity on behalf of respondents. Enumerators combined and allocated 24 one-hour tokens to activity categories after respondents narrated their previous day activities. Pilots revealed that, compared to a respondent-led allocation, this approach improved respondent recall while reducing time and cognitive burdens. Enumerators allocated tokens to the eight major activities, represented by pictures, available in the Online Appendix.^11^ Pictorial representation of activities helped illiterate respondents participate. At the end of the exercise, respondents decided whether the token allocations accurately captured activities of the previous day and enumerators made any necessary adjustments.

In comparison to traditional assisted time diaries, the Hybrid approach reduced module duration by converting respondents’ narratives into stylized time use categories, while also easing respondent burdens. Respondents were not required to aggregate time spent on different activities throughout the day or be familiar with standard clock time. While the respondent narrated her day, the enumerator recorded activities that took less than a complete hour on a separate notepad, then aggregated and rounded these inputs to activity hours. The enumerator also asked about, aggregated, and reported the total amount of time devoted to passive caregiving for each of the eight activity categories.^12^

Overall, all methods captured time use over a 24-hour time period, although the Traditional and Hybrid methods collected retrospective reports about the previous day, while the Gold Standard collected data on time spent on activities on the day they occurred. To compare data across time use methods, we assigned each of the 152 unique activity codes to one of the 8 Hybrid module categories. Table 2 in the Online Appendix describes this mapping of categories.

## 3 Study sample and experimental design

### 3.1 Study sample

Our study took place in low-income, conservative rural areas in northern Madhya Pradesh. We used the sampling frame of a randomized controlled trial spanning 197 village communities, known as gram panchayats (GPs), described in Field et al. (2021). Inclusion criteria for the sample frame were that the household had to have appeared on the payroll of India’s public workfare program (the Mahatma Gandhi National Rural Employment Guarantee Scheme, MGNREGS) in the previous year, had reported having worked for MGNREGS at some point, and had at least one married, unbanked woman. For this study, we enrolled households from 13 communities in Gwalior district.

We used household roster information to create six strata based on demographic characteristics: unmarried male respondents, unmarried female respondents, male respondents with wives under the age of 30, married female respondents under the age of 30, married male respondents with wives over the age of 30, and married female respondents over the age of 30. We stratified enrollment to study how Hybrid method performance varies across genders and life stages. Overall, we sampled 515 respondents from 212 unique households. Appendix Table A1 reports sample sizes in strata×time use method×visit cells.

Table A2 compares our respondents to rural participants in India’s second time use survey in 2019 (NSO, 2020)^13^, which was collected one year after our exercise.

Panels A, B, and C report average demographics, occupation, and time use, respectively, for each sample by gender.^14^ Individuals in our sample are younger (likely reflecting our stratified enrollment) and more likely to belong to disadvantaged “scheduled” castes and tribes. Women have similar occupations across samples: the majority (70–75 percent) report domestic duties, with 5–10 percent identifying as self-employed and day labors; only 5–8 percent are students. While most men in both samples are employed, the nature of their employment differs. In the national sample, self-employment is most common (37 percent of men) while in our sample the majority (52 percent) work as day laborers.

In terms of time use, women in both samples spend most of their waking hours occupied with chores (6 hours/day) or leisure (6–7 hours/day), with limited time devoted to market activities. Women in both samples spend similar amounts of time on paid work (1-2 h), field work (0.5 h), and active care (0.7 h). Men’s time use is also very similar across the two samples: they work for pay or in agriculture approximately 6 hours/day, do approximately 1.5 h of chores/day, and enjoy 8 h of leisure.

Overall, Table A2 shows that the patterns in time use and gender differences are similar. Thus, our sample offers a good context for understanding how the Hybrid method performs in a resource constrained setting where individuals engage in a broad range of productive and unproductive activities during the day.

### 3.2 Study design

Our validation experiment was conducted in January and February 2018.^15^ Study participants were visited thrice:

• **Visit 1-Reference Day 1:** Visit 1 served two purposes — respondent enrollment and data collection among those who enrolled. Respondents were informed that enrolling in the study would involve multiple visits that day, a return visit the next day, and a final visit in the next several weeks. Respondents were instructed not to change their daily routines, including leaving the house as usual. If they agreed, they were enrolled. Subsequently, time use for Day 1 was recorded using the Gold Standard approach.
• **Visit 2-Reference Day 1:** The next day, an enumerator visited the respondent and administered either the Traditional or Hybrid module, based on random assignment. Respondents reported on the previous day’s (reference day 1) activities, enabling within-subject comparison of the retrospective data from visit 2 against reference day 1 Gold Standard data. We also obtained basic demographic information.
• **Visit 3-Reference Day 2:** This visit occurred at least one week after visit 2. The enumerator administered one of the three time use methods, again based on random assignment stratified on community, demographic group, and method assigned in visit 2. An important motivation for visit 3 was to account for the possibility that “priming” individuals with the Gold Standard method on visit 1 improved recall during visit 2, which could influence performance differences between the Traditional and Hybrid approaches.

Respondents received small incentives (less than $1) after visits 2 and 3, and attrition was below 1%.^16^. Appendix Table A4 verifies that demographic characteristics are balanced across randomly-assigned data collection methods at visit 2 and visit 3, and Table A5 confirms Gold Standard time use for visit 2 assignments is similarly balanced.

### 3.3 Data quality monitoring

We monitored data quality throughout the validation experiment. For the Gold Standard method, this included collecting GPS data when enumerators visited a household to ensure visits were correctly spaced, and having supervisors shadow and collect parallel data through enumerator “accompaniments” for 30% of in-person surveys. Accompaniment visits tracked and provided enumerators with near real-time feedback to ensure they did not interrupt household activities. For the Traditional and Hybrid modules, we audio recorded surveys and had a different team audio audit 35% of surveys to assess protocol adherence and survey quality. Finally, 20% of second and third survey visits were “backchecked” via a separate in-person visit that asked the respondent about enumerator visits, behavior, and incentive payments.

## 4 Comparing time use modules

To validate the Hybrid method time use data, we first evaluate bias and accuracy in time use reporting, as measured by within-person mean square error relative to the Gold Standard. Next, we assess how well the Hybrid method captures the overall distribution of time use across our sample, and the extent to which the method leads to under and over-reporting on the extensive margin. Finally, we assess performance across demographic strata.

### 4.1 Activity measurement

#### A. Empirical approach

We employ within-respondent i comparisons of average time use on reference day 1. The Gold Standard-based visit v=1 forms the baseline against which we compare module performance (either Traditional and Hybrid) on v=2. We estimate:

(1) $$y_{i,v}=\beta_{1}Trad_{i,v}+\beta_{2}Hybrid_{i,v}+\delta_{i}+\epsilon_{i,v}$$

where $y_{i,v}$ is the outcome of interest (i.e. hours spent on a particular activity), $Trad_{i,v}$ and $Hybrid_{i,v}$ are dummy variables for data collected using the Traditional and Hybrid module, respectively (both variables equal 0 for v=1), $\delta_{i}$ are individual fixed effects, and $\epsilon_{i,v}$ is an error term. Standard errors are clustered at the individual level. If a given method is unbiased, we expect the coefficients on the associated method dummy to equal zero.

To create a summary test of bias that avoids over-interpreting individual coefficients, we present a $\chi^{2}$ test of joint significance of coefficients across all time use categories. We calculate this $\chi^{2}$ test using seemingly unrelated regression to account for the correlation in error terms across equations, again clustering standard errors at the individual level.

While unbiasedness is a desirable characteristic, precision matters as well. To construct a measure of misclassification that also accounts for the magnitude of reporting errors, we calculate mean square reporting error relative to the Gold Standard for both the Traditional and Hybrid approaches:

(2) $$\sqrt{\frac{1}{9}\overset{9}{\underset{n=1}{\sum }}{\left(GS_{i,n}-M_{i,n}^{m}\right)}^{2}}$$

where i is the respondent, n is each of the nine activity categories, and methods are denoted as GS for Gold Standard, and $m\in \left\{\text{Hybrid},\text{Traditional}\right\}$.

#### B. Overall assessment

**Fig. 1:**
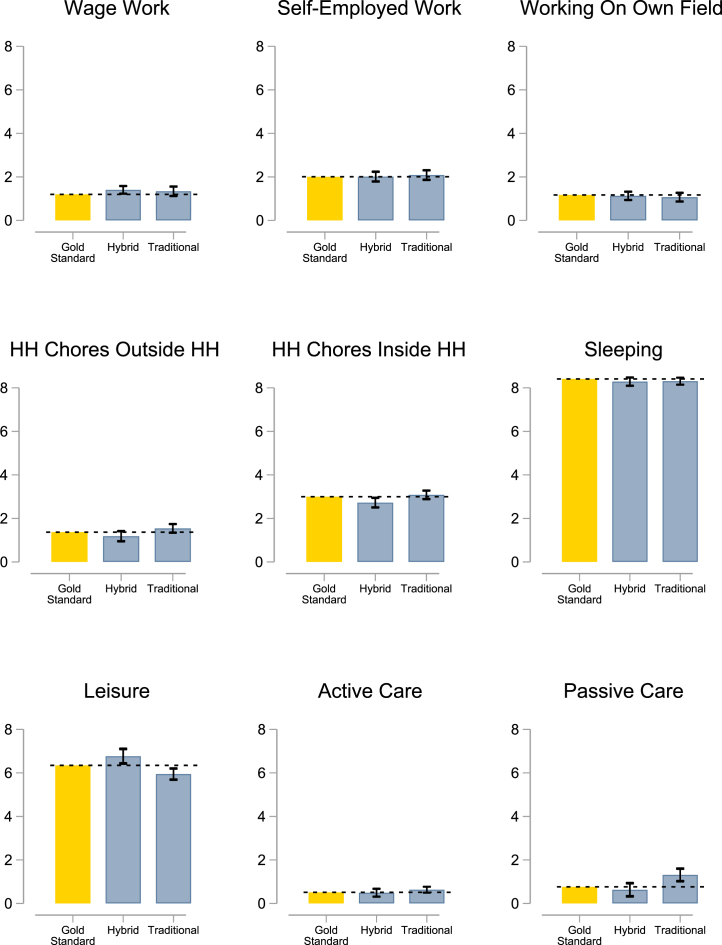
Reference day 1 module comparison. Outcomes reported in hours. The first bar in each panel represents the Gold Standard mean for the indicated outcome, and the following bars are regression-adjusted means using coefficients from Eq. (1). Sample restricted to Reference Day 1. Whiskers display 90 and 95 percent confidence intervals based on robust standard errors clustered at the individual level.

To give an overall sense of module performance, Fig. 1 graphs average time use for the eight activity categories used in the Hybrid method, along with total time spent on passive care. The first bar in each panel graphs mean time recorded per the Gold Standard. The next two bars show regression-adjusted means for the Traditional and Hybrid modules based on specification (1). The top panel in Table 2 reports point estimates and standard errors.

Overall, the Hybrid module performs well. While the joint test rejects unbiasedness at the 10 percent level (p=0.06), most differences relative to Gold Standard means are small in magnitude. (The largest significant mismeasurement is in wage work, which the Hybrid method over-reports by 0.2 h relative to a Gold Standard mean of 1.1 h). In contrast, the joint test rejects unbiasedness of the Traditional method at the 1 percent level (p=0.0003). This is driven by under-reporting of leisure, which is modest in magnitude (0.4 h relative to the Gold Standard mean of 6.3 h) and over-reporting of passive care, which is more substantial (0.55 h relative to a Gold Standard mean of 0.78 h). Both methods fare comparably in terms of root mean square error, however, which is 1.5–1.7 h relative to the Gold Standard, and slightly higher for the Hybrid method.

When interpreting our results, it is important to keep in mind that the Hybrid time use categories were developed with women in mind; thus, the performance of the Hybrid module in particular may differ by gender. Indeed, according to Table 2, both methods perform better among women (with joint tests of unbiasedness equal to 0.10 for Hybrid and 0.09 for Traditional) than men (where we reject unbiasedness at the 1 and 5 percent levels). The most problematic activity category for both the Hybrid and Traditional modules is passive caregiving: among women, the Traditional module significantly overstates passive care, while the Hybrid module significantly understates it. Both approaches significantly overstate passive caregiving among men (a 0.26–0.28 h increase relative to a 0.15 h mean per the Gold standard). This suggests that capturing simultaneous secondary activities poses a general challenge for survey-based time use data collection.

Other problematic categories for men include leisure (though deviations from the Gold Standard mean are less than 10%) and active care (overestimating by over half the Gold Standard mean for the Traditional module). The Hybrid approach underestimates chores and overestimates wage work and leisure compared to the Gold Standard activities.

Turning to root mean square error, the final column shows that both Hybrid and Traditional have comparable errors for women, while the Traditional module fares better among men, where a female-centric photo may have primed men to underreport household chores.

**Table 2:** Reference day 1 comparisons by gender.

	(1)	(2)	(3)	(4)	(5)	(6)	(7)	(8)	(9)	(10)	(11)
	Wage Work	Self Employed	Working Own Field	HH Chores Outside HH	HH Chores Inside HH	Sleeping	Leisure	Active Care	Passive Care	Joint test p-value ($\chi^{2}$)	Root MSE
*Full Sample*											

$\beta_{1}$: Traditional Module	0.145	0.077	−0.102	0.172	0.085	−0.103	−0.399***	0.124	0.549***	0.000***	
	(0.120)	(0.122)	(0.111)	(0.110)	(0.109)	(0.089)	(0.142)	(0.075)	(0.160)		
$\beta_{2}$: Hybrid Module	0.210**	0.007	−0.041	−0.188	−0.271**	−0.122	0.422**	−0.016	−0.134	0.061*	0.179**
	(0.097)	(0.124)	(0.105)	(0.129)	(0.122)	(0.105)	(0.186)	(0.100)	(0.168)		(0.097)
Dependent Var Mean	1.098	1.973	1.150	1.387	3.105	8.463	6.309	0.514	0.781		1.541
N	998	998	998	998	998	998	998	998	998		499

*Females*											

$\beta_{1}$: Traditional Module	−0.002	0.106	−0.039	0.105	0.130	−0.102	−0.299	0.101	0.851***	0.087*	
	(0.079)	(0.147)	(0.121)	(0.156)	(0.183)	(0.128)	(0.211)	(0.131)	(0.309)		
$\beta_{2}$: Hybrid Module	0.025	−0.093	0.052	−0.089	0.097	−0.207*	0.296	−0.082	−0.556*	0.098*	0.025
	(0.117)	(0.159)	(0.070)	(0.152)	(0.174)	(0.123)	(0.220)	(0.168)	(0.316)		(0.133)
Dependent Var Mean	0.314	1.655	0.474	1.244	5.241	8.584	5.694	0.794	1.425		1.654
N	494	494	494	494	494	494	494	494	494		247

*Males*											

$\beta_{1}$: Traditional Module	0.287	0.049	−0.162	0.237	0.042	−0.103	−0.495***	0.146*	0.256***	0.012**	
	(0.223)	(0.194)	(0.185)	(0.155)	(0.120)	(0.124)	(0.190)	(0.077)	(0.090)		
$\beta_{2}$: Hybrid Module	0.393**	0.106	−0.133	−0.285	−0.637***	−0.037	0.546*	0.049	0.283***	0.000***	0.330**
	(0.154)	(0.192)	(0.198)	(0.208)	(0.166)	(0.170)	(0.300)	(0.109)	(0.106)		(0.141)
Dependent Var Mean	1.866	2.285	1.813	1.527	1.012	8.344	6.912	0.240	0.150		1.431
N	504	504	504	504	504	504	504	504	504		252

Column headers for 1–9 denote variable outcomes, reported in hours. Sample includes Reference Day 1 visits only. All regressions are as specified in Eq. (1), including individual fixed effects. Standard errors clustered at individual level in parentheses. Column 10 indicates the p-value from an $\chi^{2}$ test that the coefficients across all categories are jointly equal to zero, evaluated using seemingly unrelated regression on individually demeaned data; standard errors for the joint test are similarly clustered at the individual level. Column 11 reports coefficient for Hybrid method in individual-level regression on square root of sum of squared difference from visit 1 Gold Standard time, with dummies for strata. Standard errors for column 11 are robust. Dependent variable mean in columns 1–9 is for the Gold Standard Day 1 visit; in column 11, the dependent variable statistic reports the Traditional method value for the outcome variable. *p<0.10, **p<0.05, ***p<0.01.

#### C. Distributional comparisons

**Fig. 2:**
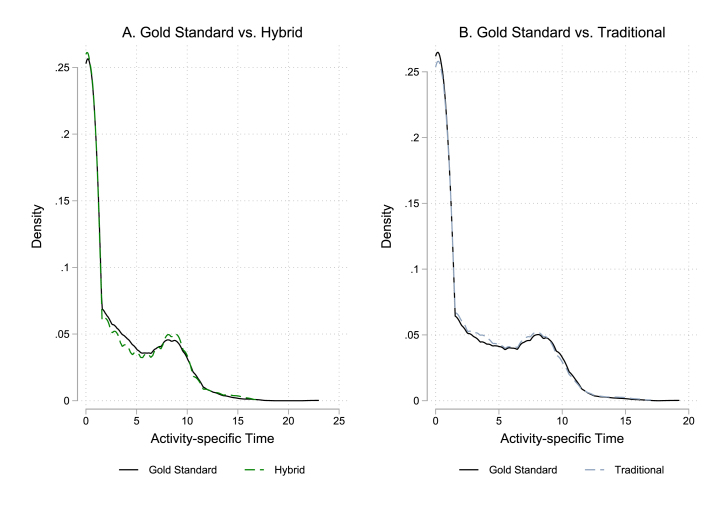
Distribution of time use on reference day 1 by data collection method. Time distribution plotted over 4,500 activity-category observations across 250 respondents assigned Traditional method on visit 2, and 4,482 activity-category observations across 249 respondents assigned Hybrid method for visit 2.

Researchers may be interested in more than average time use. To assess distributional performance, Fig. 2 reports kernel densities of time use by method. To save space, we first transform the data to be at the respondent×activity category level; thus, the plots report relative frequency of different activity time allocations without distinguishing between activities. To facilitate within-person comparisons, Panel A limits the sample to individuals assigned the Hybrid method on visit 2, while Panel B limits the sample to individuals assigned the Traditional method.

Overall, the distributions are strikingly similar, with both methods doing well capturing long-duration activities. The Hybrid method, on the other hand, appears to under-count lower duration activities; this could be due to rounding errors and challenges associated with enumerators assigning short increments of time to one-hour tokens. The density plots also indicate potential differences in the rate of reporting no time at all on a given activity.

To investigate these issues more formally, we return to reference day 1 and create two activity-level measures of method performance *relative* to the Gold Standard. First, to measure extensive margin over-reporting, we consider activities for which time spent per the Gold Standard is zero and construct a dummy equal to one when method m records positive time spent on a. Second, to measure under-reporting, we consider activities where the Gold Standard records positive time spent and construct a dummy variable equal to one when method m fails to record time on a.

We stack the data at the individual i × activity a × method m ∈{Hybrid,Traditional} level and estimate:

(3) $$y_{i,a,m}=\alpha_{1}Hybrid_{i,m}+\zeta_{a}+\psi_{s}+\epsilon_{i,a,m}$$

where $y_{i,a,m}$ is the outcome of interest and $Hybrid_{i,m}$ is an indicator variable for whether individual i was administered the Hybrid method on visit 2. $\zeta_{a}$ are activity category fixed effects, $\psi_{s}$ are strata fixed effects, and $\epsilon_{i,a,m}$ is an error term clustered at the individual level. The omitted group is the set of respondents randomly assigned to receive the Traditional module during visit 2, so $\alpha_{1}$ should be interpreted as the difference in reporting error relative to the Traditional module. We examine results separately for men and women.

Results are in Table 3. Column (1) investigates over-reporting, by limiting attention to zero-duration activities per the Gold Standard. Here, the Hybrid method has similar advantages for both genders and is 4–6 percentage points less likely to over-report relative to the Traditional module. The latter erroneously reports positive time for 14–15 percent of zero duration activities.

In columns (2)–(4), we study under-reporting relative to the Gold Standard for low, medium, and high duration activities (classified based on terciles of Gold Standard duration, excluding zero responses).^17^ Downward censoring of activity categories may be an issue with the Hybrid module since categories must be grouped in one hour intervals, and therefore may be more likely to miss activities of short duration. Among both genders, the Traditional module under-reports low-duration activities 22 percent of the time, while underreports for medium- and high-duration activities are rare. While Hybrid-Traditional differences are muted among women, the Hybrid module is 25 and 13 percentage points more likely to underreport low and medium duration activities respectively among men. Columns (5)–(8) turn to root mean square error to assess the magnitude of these reporting errors, which penalize larger errors more. Again, for women Hybrid-Traditional differences are relatively more minor, with one significant positive difference for medium duration activities (approximately 25 percent the size of the Traditional error for this category), and the remaining coefficients negative, but statistically insignificant. Among men, however, the Hybrid method is associated with increased errors for both low and medium duration activities, at over half the size of the Traditional errors for men in each case. In short, the Hybrid module performs better for females, for whom the categories were originally designed, than for male respondents.

**Table 3:** Hybrid performance by duration of activities.

	Overreport	Underreport			Root Mean Squared Error vs. Gold Standard			
	(1)	(2)	(3)	(4)	(5)	(6)	(7)	(8)
	GS=0	GS=Low	GS=Mid	GS=High	GS=0	GS=Low	GS=Mid	GS=High
*Females*								

$\alpha_{1}$: Hybrid Module	−0.041*	0.035	0.045**	0.004	−0.056	−0.037	0.380***	−0.113
	(0.021)	(0.040)	(0.021)	(0.007)	(0.064)	(0.097)	(0.139)	(0.172)
Traditional Mean	0.135	0.217	0.048	0.006	0.275	1.049	1.512	1.440
N	767	403	456	350	767	403	456	350

*Males*								

$\alpha_{1}$: Hybrid Module	−0.056**	0.252***	0.130***	0.000	−0.121	0.618***	0.876***	0.215
	(0.022)	(0.041)	(0.030)	(0.016)	(0.078)	(0.148)	(0.227)	(0.193)
Traditional Mean	0.147	0.216	0.058	0.025	0.376	0.966	1.550	1.549
N	905	373	344	394	905	373	344	394

The top column headers denote variable outcomes, and the second level specifies the sample included for that regression, based on Gold Standard Visit 1 reports *(GS)*. Outcomes in columns 5 through 8 are reported in hours. Gold Standard sample restriction in columns 2 - 4 and 6 - 8 are terciles after excluding reports for that respondent-activity category equal to 0. Sample restricted to Reference Day 1. All regressions include strata and activity category fixed effects. Standard errors clustered at individual level in parentheses. *p<0.10, **p<0.05, ***p<0.01.

#### D. Differences across demographic groups

The stark gender differences in under-reporting associated with the Hybrid method suggests that under-reporting is caused by more than just “structural” issues, such as assigning low-frequency activities to one-hour increments. Rather, our female-centric categories may have made accurately capturing men’s time use harder. To test this hypothesis we decompose results by life stage, by estimating Eq. (1) within each of our six demographic strata. Tables A6 and A7 report results, with the $\chi^{2}$ joint test in column (10), and Hybrid-Traditional differences in root mean squared error in column (11).

Within these demographic strata, we cannot reject the joint null for Hybrid module coefficients for older married women and married men. While passive care is captured accurately for most demographic groups, the less detailed Hybrid module still under-captures young married women’s time on this activity. These women spend nearly 3.5 h per day taking care of family members (largely children) while undertaking other activities. Hybrid underreporting may reflect women’s tendency to under-report or recognize such care as it occurs.

When root mean squared error is considered, Table A7 shows that the Hybrid method only under-performs the Traditional module for young unmarried men. This is because the Hybrid method mis-classifies chores as leisure for this group.^18^ This could be due to social desirability bias, for example, if young men fail to mention chores when describing their day in broad strokes, or if they pushed back against the number of tokens initially allocated to household work. The photos used to denote chores included a focal female, and may have exacerbated desirability bias, emphasizing the importance of carefully selecting images for activity classification.

Finally, one of our goals for the Hybrid approach was to find a way to capture time use that works well with low-literacy populations that would be encountered in a national survey in a low-income setting. In our sample, for example, 28 percent of respondents said they had never attended school. Figure A1 in the Appendix summarizes differences in root mean square error by whether respondents had any schooling. We find no significant differences between methods for both those with and without education. Thus while the Hybrid does not outperform the Traditional approach for uneducated respondents, it also does not fare any worse.

### 4.2 Robustness and threats to internal validity

Our findings show that the Hybrid method captures time use as well as the Traditional assisted diary in nine broad activity categories. In comparison to the Traditional module, respondents are less likely to over-report time spent on zero duration activities, although men, in particular, tend to under-report activities more generally. We now evaluate whether these conclusions are robust to general priming concerns, potential intrahousehold interactions, and gender dynamics.

#### A. Respondent priming

A first concern is that participating in Gold Standard surveys on visit 1 may have affected respondents’ recall and reporting during visit two. Insofar as Gold Standard surveys improved recall, this overstates the performance of both the Traditional and Hybrid methods (perhaps to differing degrees) and may make it more difficult to identify performance differences between the two methods.

In order to evaluate this risk, we randomly assigned respondents to one of the three time use methods during a third visit. This allows us to evaluate performance without Gold Standard priming on the reference day. That said, taking part in the Gold Standard on visit 1 might make respondents persistently more aware of how they spend their time – potentially in anticipation of further visits – which would lead us to overstate method performance and underreject method differences even on Reference Day 2. Since all respondents were administered the Gold Standard on visit 1, we cannot directly test this hypothesis. We can, however, indirectly test it under the assumption that priming effects dissipate over time.

Our priming test compares performance of the methods compared to the Gold Standard time allocations on reference day 1 versus reference day 2. The above concerns suggest differences from the Gold Standard should be larger for reference day 2 relative to reference day 1.

Our main results relied on within-person comparisons of the Traditional and Hybrid methods (visit 2, reference day 1) to the Gold Standard (visit 1, reference day 1). An ideal relative performance assessment would hold sample sizes and estimation techniques constant across the two reference days. However, a within-person comparison is not possible for reference day 2 – we only conducted one visit and during this visit, one third of the sample was randomized to each of the three time use methods. In order to generate an “apples-to-apples” comparison, we therefore rely on a bootstrap-style procedure with 500 replications to produce cross-person comparisons for both reference days.

Sampling works as follows: for reference day 2 we randomly select (with replacement) 21 observations from each treatment group × strata cell.^19^ For reference day 1, visit 2, we randomly select (with replacement) 21 observations from each treatment group × strata cell. For reference day 1 visit 1, we select 21 observations per stratum from the group assigned the Traditional method on visit 2, and another 21 observations per stratum from the group assigned the Hybrid method on visit 2. For each reference day, we limit the sample to either Hybrid+Gold Standard or Traditional+Gold Standard observations and estimate:

(4) $$y_{i}=\beta_{0}+\beta_{1}Meth_{i}+\psi_{s}+\epsilon_{i}$$

$y_{i}$ is a time allocation, $Meth_{i}$ is a dummy variable denoting either Traditional or Hybrid method, $\psi_{s}$ is a vector of strata dummies, and $\epsilon_{i}$ is a heteroskedasticity robust error term. For reference day 1, regressions combine Hybrid observations with Gold Standard observations *that were assigned to Traditional on visit 2* (and vice versa) – this ensures samples assigned to each treatment group remain mutually exclusive and we focus exclusively on these across-person comparisons to assess priming.

For each replication we record two summary measures of performance relative to the Gold Standard, analogous to our earlier focus: first, the p-value from a $\chi^{2}$ test that $\beta_{1}$ is jointly equal to zero across all 9 time use categories; second, the root mean sum of squared $\beta_{1}$ coefficients across the 9 categories, which assess accuracy compared to the Gold Standard. Higher p-values and lower root mean sums of squares indicate better performance.

**Fig. 3:**
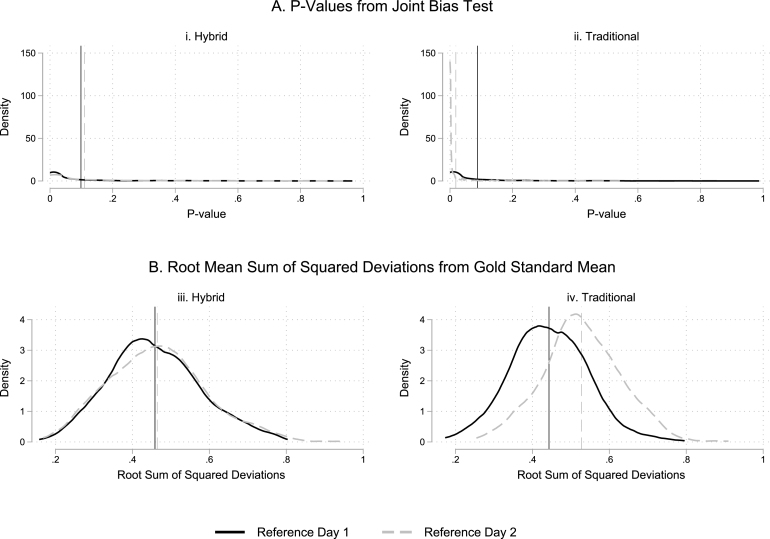
Traditional and Hybrid module performance across reference days 1 and 2. Panel A graphs the distribution of p-values from a joint $\chi^{2}$ test of bias across 500 bootstrap replications. Panel B graphs the sum of squared differences relative to the Gold Standard across all 9 time use categories depicted in Table 2. Vertical lines denote mean values across all replications. Each replication resamples 21 observations from each treatment group × demographic strata × reference day cell, ensuring sample sizes in each treatment group on each reference day are identical.

Fig. 3 plots the distribution of these performance measures across 500 replications, by reference day and treatment. The solid black lines show that on reference day 1, performance of the Hybrid and Traditional approaches are roughly equivalent, mirroring our main analysis. However, performance diverges on reference day 2 (dashed gray lines). While the Hybrid method’s performance remains roughly constant across reference days, the Traditional method performs noticeably worse. This suggests priming had a greater “boosting” effect on the Traditional module’s performance; this makes sense, because both the Traditional and Gold Standard aim to map activities into a very detailed set of categories; doing so on Gold Standard visit 1 may have helped respondents provide the required detail on visit 2. This investigation suggests that our main analysis likely underestimates the benefits of the Hybrid module, which may outperform the Traditional approach once priming is fully accounted for.

#### B. Intrahousehold interactions and reporting

Another source of concern is that multiple household members were interviewed: our sample included 499 respondents from 212 households.^20^ This means that two members of the same household may have received different survey methods in visit 2 or 3. In practice, roughly two-thirds of visit 2 respondents had a household member who participated in the study and was administered a different method, and between 43 and 48 percent of visit 3 respondents had a household member who was administered a different method.

We attempted but were unable to guarantee complete privacy during an interview. As a result, another member’s interview could have affected the respondent’s report. While household structure (and thus the number of other household members enrolled) is not random, the time use methods assigned to other household members are. To ascertain whether household spillovers influence our estimates of method performance, we ask whether methods assigned to other household members on the same day influence time use reports. To accomplish this, we take the approach of specification (1), regressing time use on individual-specific method dummies, individual fixed effects (which absorb household structure), and indicators for whether another household member received each of the time use methods on visit day 2. Appendix Table A8 reports results. Our joint tests, reassuringly, fail to reject the null hypothesis that other household members’ method assignments had no effect on an individual’s own report, and Hybrid module results look very similar to the results in the unaugmented regressions.

#### C. Surveyor gender and respondent reports

Another factor to consider is whether the gender of the enumerators influences respondent reports, especially since the Hybrid approach shows that younger men are less likely to report on household chores. Throughout the validation study, we rotated enumerators across respondents in order to optimize logistics; in practice, respondents were administered surveys by enumerators of a different gender just under half the time; thus, we have ample power to study gender interaction effects.

To assess, we examine differences on reference day one for respondents by method and gender of their assigned enumerator. We augment specification (1) to include a “different surveyor gender on visit” dummy and its interaction with the Traditional and Hybrid dummies. Appendix Tables A9 and A10 show results separately for male and female respondents. The joint test on the coefficient for surveyor gender indicates that, overall, the gender of the surveyor did not affect time allocations during the Gold Standard method. Moreover, we find no evidence of systematic differences in reporting under the Hybrid or Traditional approaches due to surveyor gender.

### 4.3 Cost and implementation considerations

Finally, we compare the cost and ease of implementation of the Hybrid and Traditional modules. We are less interested in Gold Standard comparisons because it is resource intensive and ill-suited to wide-scale use.

**Fig. 4:**
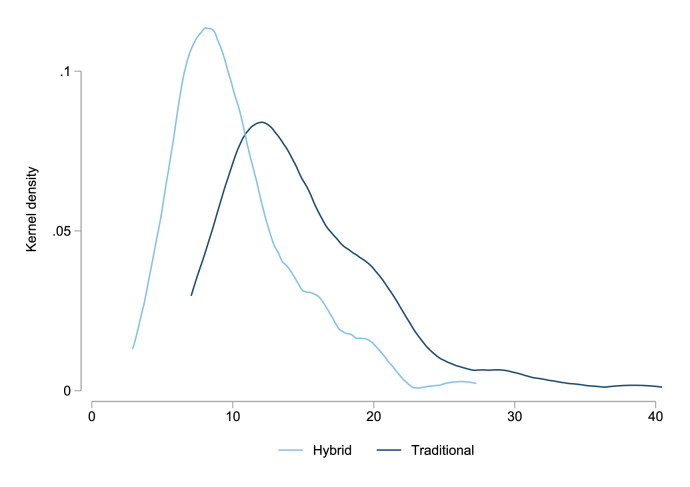
Distribution of survey duration by method. Figure shows kernel density plot of survey duration. Sample restricted to Reference Day 2. p-values from K-Smirinov test that Traditional method distribution = Hybrid method distribution = 0.000. The p-value from a t-test for differences in mean duration by method = 0.000.

The Hybrid method is cost and implementation effective. Fig. 4 plots survey duration for reference day 2 for the Traditional and Hybrid modules; the Hybrid’s time distribution indicates significant time savings (the p-value from a Kolmogorov–Smirnov test rejects equality of distributions at the 0.01 level). Average completion time for the Traditional module was 14 min, while the Hybrid method took 9 min.^21^ The shorter Hybrid duration likely reflects multiple features of the module, including the need for enumerators to classify time across only 8 categories, compared to 152 in the Traditional module, and the Hybrid module’s focus on hourly intervals, in contrast to up to the Traditional module’s capture of up to 6 activities per hour in minute increments. For each Traditional activity reported, enumerators also needed to select the activity location and whether the activity was paid, which increased the time required to administer the module, particularly for respondents with many short-duration activities. Likely reflecting this, time differences in module implementation are higher in the right tail: For example, at the 90th percentile, the difference is 6 min, and it is 8 min at the 95th percentile. An average time savings of 5 min may seem modest, but it is a 33 percent reduction in module time. In the context of a long, multi-module survey, this could confer significant cost savings or the ability to add a new survey module.

Cost savings are amplified by the fact that training enumerators on the Hybrid module takes less time. The Traditional method required extensive training to ensure that enumerators could categorize time use into 152 distinct activity categories. Three full days of training were required to properly prepare enumerators to classify these activities, as well as additional refresher training to clarify questions. The Hybrid method, on the other hand, required only one day of training to ensure enumerators could easily and correctly categorize the eight activity codes. Reduced training time and approaches that work better with less skilled enumerators are both valuable in large scale surveys.

Table 4 presents cost estimates that highlight this savings in enumerator training and implementation time. Using input costs from our field work, we estimate that a 100 respondent standalone time use survey using the Hybrid approach would cost approximately $7 per survey, or 62% of the $11 per survey cost for the Traditional method.^22^ When implemented at scale with a hypothetical respondent pool of 10,000, economies of scale mean that survey costs fall to $4.39 per respondent for the Hybrid module, 77% as high as the $5.68 per respondent for the Traditional approach. The cost difference between modules is partly due to the extensive field team training needs when collecting data using the Traditional approach. Moreover, relative time savings in administering the Hybrid module are such that the variable (non-training) components to Hybrid data collection are 80% of variable costs incurred for the Traditional module.

**Table 4:** Time use survey module costs.

	Training cost	Surveying cost	Monitoring cost	Total	Cost per survey module	Cost as proportion of traditional module
*n = 100 respondents*						
Gold Standard	$742	$798	$78	$1,618	$16.18	1.47
Traditional	$461	$585	$58	$1,104	$11.04	1.00
Hybrid	$165	$471	$47	$682	$6.82	0.62

*n = 10,000 respondents*						
Gold Standard	$3,160	$64,105	$7,446	$74,711	$7.47	1.32
Traditional	$3,160	$48,041	$5,588	$56,789	$5.68	1.00
Hybrid	$1,064	$38,370	$4,464	$43,898	$4.39	0.77

Costs in USD using 64 INR to 1 USD, consistent with exchange rates at the time of the survey. Training costs include costs for training venue, training materials, refreshments, enumerator and field team salaries for training days. We assume the Gold Standard and Traditional method require 3 days of training and a refresher training, whereas the Hybrid requires a 1 day training. Surveying costs include field transportation, enumerator salaries, supervisor salaries, laptop rentals for supervisors, tablet rentals for enumerators, for teams of 5 enumerators and 2 supervisors for the small n survey, and 25 total enumerators and 5 supervisors for the large n survey. Monitoring costs include travel and tablet rental for supervisors conducting survey backchecks. We assume Gold Standard enumerators can complete 3 surveys per day, Traditional method 4, and Hybrid method 5, when accounting for time to locate and enroll respondents in the survey. Additional cost assumption details are available in the Appendix.

These cost estimates exclude one up-front cost of the Hybrid module: initial scoping work to select Hybrid categories, and then photo selection and category refinement through field visits and practice surveys. Appropriate selection of both depends on the respondent population, and our results highlight this should be done carefully. In practice, our categorization and photo selection, along with pre-piloting activities that assessed how well detailed time activities mapped to our selected categories, took six work days, including three full in-field days, with a Research Associate and two skilled supervisor-level field staff. These staffing and associated costs totaled approximately $500. We have excluded these costs from the table since they may vary substantially depending on size and scope of the survey, and heterogeneity of the respondent population.

Aside from cost savings, feedback from field staff indicate that the Hybrid module was significantly easier to administer in the field. The cards with photos and tokens encouraged respondents to interact with the method, and the lack of extensive probing and relative brevity reduced respondent and enumerator fatigue. In contrast, the detailed probing required to classify activities sometimes taxed respondents administered the Traditional (and Gold Standard) modules. The most complicated part of the Hybrid method for enumerators was calculating “left over” minutes. If respondents reported an activity for 30 min in the morning, for example, and then 20 min later in the day, the enumerator needed to set aside these minutes and then add them up at the end of the exercise to determine whether to allocate an hour to that activity or not.

## 5 Discussion

The Hybrid time use module, in which respondents narrate their days and enumerators assist respondents in allocating time to a limited number of stylized time use categories, accurately captures most respondents’ average time use in a poor, rural setting. While we reject unbiasedness relative to the Gold Standard at the 10 percent level, deviations from Gold Standard means are small. After accounting for priming, the Hybrid appears to perform quite well compared to a widely used assisted retrospective diary approach, which also suffers from bias relative to the Gold Standard. The Hybrid method has the advantage of being relatively inexpensive and requiring less time to administer and to train enumerators, an attractive feature for researchers who wish to limit respondent fatigue and cognitive burden.

The Hybrid approach requires the researcher to identify a concise set of activity categories and does not capture *when* activities occur during the reference period activities. And while it is effective in capturing *average* time use across activity categories, it is more likely to miss short duration activities on the extensive margin.^23^ Given this, more traditional approaches would be better suited for research that requires substantial detail on how respondents spend their days. In comparison, the Hybrid approach is likely to be particularly valuable as an additional module in large-scale household surveys which are repeated and seek to measure changes in work and leisure patterns as economies develop.

Based on relative performance across demographic groups, we believe that the Hybrid method requires careful category and visual aid selection to avoid inducing desirability response in time reports. To maximize data quality, it is important for the researcher to pilot the Hybrid approach and carefully select activity categories and photos used to depict them. Because time use varies across populations and demographic groups, we recommend that interested researchers first conduct targeted qualitative work to understand how their study population spends its time before developing Hybrid categories.

Our randomized validation design is informative for researchers who want to test novel approaches to collecting time use data in resource-constrained settings. Additional testing of the Hybrid approach in other settings would build an understanding of the extent to which our findings are externally valid. Another important research avenue would be to combine a version of the Hybrid method with approaches from psychology designed to understand respondents’ perceptions of well-being as they engage in specific activities, in line with experiential time use approaches, as in Kahneman et al. (2004).

Ultimately, we hope this paper highlights the feasibility of systematically incorporating more high-quality time use data collection into major national surveys in emerging economies. Doing so, for example, would significantly improve researchers’ ability to understand how households’ labor allocation – particularly that of women – evolves over the course of economic development, and how this is shaped by contextual factors like institutions and social norms.

## Data Availability

Replication code was attached.

## Appendix A Supplementary data

MMC S1 Additional Tables and Figures can be found in Online Appendix A.

MMC S2 .

## Footnotes

1 As a recent example, Jagnani (2022) uses the 1998–1999 Indian Time Use Survey to estimate how the number of hours a child spends sleeping impacts human capital acquisition.

2 Research by Daum et al. (2019) suggests these barriers can be overcome through significant training of potential respondents.

3 A recent paper by Seymour et al. (2020) makes significant improvements on previous approaches to quantify the value of different time use methods, testing approaches across multiple study settings, but lack of experimental variation precludes the authors from drawing the types of conclusions that we can here.

4 Daum et al. (2019) demonstrate that rural Zambian respondents were able to regularly enter data in a custom designed, picture-based app. They document significant differences in app-based time use compared to a traditional 24-hour recall, which they argue point to reporting bias, but do not compare results to a gold standard. In contrast, we trialled a phone-based approach entailing calling respondents at multiple intervals and found that some respondents tended to forget to carry the phone and frequently did not respond to phone calls to collect time use data. .

5 This was the last official time use survey conducted prior to our experiment; the next official time use survey was conducted in 2019, after our experiment was completed.

6 Additional details on the protocols and scripts are available in the Online Appendix.

7 There are no significant differences across time use methods in likelihood of leaving home, suggesting respondents went about their normal activities when this method was administered.

8 Using pictures and physical allocation to represent time draws upon activities described by Narayan-Parker (1996). This method is also similar to one used in some countries in the 2016 Young Lives Survey, in which respondents were asked to allocate 24 pebbles to activity categories, but without the help of PRA-inspired visual aids.

9 This pilot used a standard observation-based approach, a modified version akin to our Gold Standard, a mobile phone-based approach, and the Traditional method.

10 An important consideration in employing photos for this purpose is that the images do not overly restrict respondents’ interpretation of the category components.

11 Additional category descriptions were provided to enumerators but not read aloud to respondents as they both took longer to explain and were not necessary since respondents directly narrated previous day activities. These additional categories are also described in the Online Appendix.

12 In practice, this amounted to enumerators asking respondents if they had responsibility for a dependent for whom no one else was caring for after each described activity.

13 This is also India’s first nationally-representative survey; the survey in 1998/99 was undertaken as a pilot.

14 Occupation categories do not add up to 100 percent because we omit indicators for the unemployed and unpaid helpers, each 5 percent or less in both data sources.

15 Enumerator scripts are available in the Online Appendix.

16 Of the 515 individuals enrolled, 499 completed visits 1 and 2, and 497 completed visit 3.

17 Low duration activities last up to 2.89 h, medium duration activities last 2.9 to 7 h, and high duration activities exceed 7 h.

18 Chores in the home are under-estimated by 1.6 h, which is substantial compared to a mean of 2.2 h per the Gold Standard, outdoor chores are unederestimated by 0.8 h compared to the Gold Standard mean of 2.2 h, and leisure is overestimated by 2.1 h relative to a Gold Standard mean of 7.5 h.

19 The smallest treatment group × strata cell size in our data is 21.

20 18% of households had one member enrolled, 48% two members, and the remaining 34% three or more.

21 We focus on reference day 2 since reference day 1 surveys included an additional demographic background module.

22 Additional cost details are available in the Appendix in figures A2 and A3.

23 On the other hand, the Hybrid module is less likely to generate “false positives”, i.e., record time in a high-level activity the respondent did not actually undertake.
