# Supplementary material for: Measuring time use in rural India: Design and validation of a low-cost survey module
Source: J Dev Econ. 2023 Sep;164:103105. doi: 10.1016/j.jdeveco.2023.103105 (PMC10423985; doi:10.1016/j.jdeveco.2023.103105)
Supplement: MMC S1 — Additional Tables and Figures can be found in Online Appendix A. [file mmc1.pdf]

A. Appendix

A.1. Additional Tables and Figures

Table A1: Sample Sizes by Strata, Visit, and Method Assignment

**Category**	**N**		
Married Women 16-30	87		
Married Women 30+	86		
Unmarried Women 16-30	74		
Man married to woman 16-30	87		
Man married to Woman 30+	86		
Unmarried Man 16-30	79		
**Visit 1**	**Gold Standard**		
Full Sample	499		
**Visit 2**	**Traditional**	**Hybrid**	
Married Women 16-30	42	45	
Married Women 30+	44	42	
Unmarried Women 16-30	37	37	
Man married to woman 16-30	45	42	
Man married to Woman 30+	43	43	
Unmarried Man 16-30	39	40	
**Visit 3**	**Traditional**	**Hybrid**	**Gold Standard**
Married Women 16-30	31	27	29
Married Women 30+	26	28	32
Unmarried Women 16-30	21	31	22
Man married to woman 16-30	28	27	30
Man married to Woman 30+	29	29	28
Unmarried Man 16-30	30	24	25

Table A2: Respondent Characteristics Compared to National Time Use Survey Respondents

	Females				Males					
	Experimental Sample		National Time Use Sample		Experimental Sample		National Time Use Sample			
	(1)	(2)	(3)	(4)	(5)	(6)	(7)	(8)	(9)	(10)
	Mean	N	Mean	N	Difference	Mean	N	Mean	N	Difference
Panel A: Demographics										
Married	0.668	247	0.760	109608	0.092	0.679	252	0.711	108384	0.033
	(0.472)		(0.427)		[1.676]	(0.468)		(0.453)		[1.758]
Age	29.182	247	37.587	109608	8.405	32.556	252	37.792	108384	5.236
	(11.721)		(14.607)		[57.275]	(13.598)		(14.888)		[57.739]
Scheduled Caste or Scheduled Tribe	0.466	247	0.328	109608	-0.138	0.464	252	0.331	108384	-0.133
	(0.500)		(0.469)		[1.840]	(0.500)		(0.471)		[1.826]
Other Backward Caste	0.481	231	0.430	109608	-0.051	0.498	243	0.424	108384	-0.074
	(0.501)		(0.495)		[2.007]	(0.501)		(0.494)		[1.952]
Num. household members	5.722	245	4.455	109608	-1.268	5.385	252	4.494	108384	-0.891
	(2.091)		(1.959)		[7.714]	(2.302)		(1.914)		[7.422]
Num. household members < age 16	1.911	247	0.600	109608	-1.311	1.635	252	0.548	108384	-1.087
	(1.454)		(0.905)		[3.547]	(1.432)		(0.870)		[3.374]
Above Primary Education	0.429	247	0.408	109608	-0.021	0.738	252	0.579	108384	-0.159
	(0.496)		(0.491)		[1.927]	(0.441)		(0.494)		[1.914]
Panel B: Occupations										
Domestic Duties	0.750	240	0.705	109608	-0.045	0.020	248	0.116	108384	0.096
	(0.434)		(0.456)		[1.813]	(0.141)		(0.320)		[1.252]
Self Employed	0.087	240	0.054	109608	-0.033	0.222	248	0.373	108384	0.151
	(0.283)		(0.226)		[0.899]	(0.416)		(0.484)		[1.891]
Day Laborer	0.096	240	0.086	109608	-0.010	0.524	248	0.278	108384	-0.246
	(0.295)		(0.281)		[1.116]	(0.500)		(0.448)		[1.751]
Wage Worker	0.004	240	0.030	109608	0.026	0.056	248	0.116	108384	0.060
	(0.065)		(0.172)		[0.684]	(0.231)		(0.321)		[1.254]
Student	0.050	240	0.078	109608	0.028	0.169	248	0.105	108384	-0.065
	(0.218)		(0.268)		[1.066]	(0.376)		(0.306)		[1.198]
Panel C: Time Use (Experimental Sample Limited to Subset Randomly Selected for Traditional Method on Visit 2)										
Wage Work	0.435	124	0.564	109608	0.128	2.176	125	2.353	108384	0.177
	(1.750)		(1.827)		[10.117]	(4.024)		(3.510)		[19.338]
Self Employed	1.540	124	0.371	109608	-1.170	2.504	125	1.710	108384	-0.794
	(1.998)		(1.148)		[6.356]	(3.419)		(2.768)		[15.249]
Working Own Field	0.540	124	0.463	109608	-0.078	1.648	125	1.425	108384	-0.223
	(1.679)		(1.452)		[8.040]	(3.043)		(2.563)		[14.120]
HH Chores Outside HH	1.129	124	1.782	109608	0.653	1.056	125	1.243	108384	0.187
	(1.281)		(1.824)		[10.097]	(2.092)		(2.101)		[11.574]
HH Chores Inside HH	5.161	124	4.065	109608	-1.097	0.216	125	0.387	108384	0.171
	(2.827)		(2.182)		[12.083]	(0.667)		(1.105)		[6.090]
Active Care	0.774	124	0.736	109608	-0.038	0.280	125	0.210	108384	-0.070
	(1.209)		(1.325)		[7.334]	(1.202)		(0.610)		[3.362]
Leisure	5.919	124	6.900	109608	0.980	7.824	125	7.639	108384	-0.185
	(3.130)		(2.555)		[14.149]	(4.137)		(2.876)		[15.844]
Sleep	8.500	124	9.120	109608	0.620	8.296	125	9.034	108384	0.738
	(1.266)		(1.667)		[9.231]	(1.832)		(1.605)		[8.842]

Source: Madhya Pradesh time use survey and 2019 Indian Time Use Survey. In Panel C, the experimental sample time use is reported for visit 2, only for those who were assigned the traditional method. Indian time use survey data restricted to rural individuals between the ages of 16 and 70, in line with experimental sample. Indian time use survey data weighted to be nationally representative. Standard deviations in parentheses.

Table A3: Respondent Demographic Characteristics by Gender

	Unmarried 16-30			Married Under 30			Married Over 30		
	(1) Female Mean	(2) Male Mean	(3) Difference	(4) Female Mean	(5) Male Mean	(6) Difference	(7) Female Mean	(8) Male Mean	(9) Difference
Married[†]	0.000	0.038	-0.038*	0.943	0.954	-0.011	0.965	0.988	-0.023
			(0.022)			(0.034)			(0.023)
Age	18.054	19.329	-1.275***	25.908	29.138	-3.230***	42.070	48.163	-6.093***
			(0.367)			(0.808)			(1.402)
Scheduled Caste household	0.493	0.405	0.087	0.390	0.390	-0.000	0.463	0.463	-0.000
			(0.083)			(0.077)			(0.078)
Scheduled Tribe household	0.030	0.063	-0.033	0.073	0.073	0.000	0.049	0.049	0.000
			(0.035)			(0.041)			(0.034)
Other Backward Caste household	0.448	0.506	-0.059	0.512	0.512	0.000	0.476	0.476	0.000
			(0.083)			(0.079)			(0.078)
Num. household members	5.865	4.392	1.472***	6.184	6.471	-0.287	5.119	5.198	-0.079
			(0.279)			(0.368)			(0.313)
Num. household members < age 16	1.703	0.835	0.867***	2.471	2.437	0.034	1.523	1.558	-0.035
			(0.195)			(0.217)			(0.207)
Num. household members < age 7	0.392	0.089	0.303***	1.218	1.195	0.023	0.465	0.453	0.012
			(0.093)			(0.165)			(0.113)
Household Income	5872.917	7657.971	-1785.054	6904.762	7084.756	-179.994	6604.573	6604.573	-0.000
			(1295.090)			(1314.248)			(1132.885)
Years of Education	8.054	10.215	-2.161***	4.563	7.989	-3.425***	0.855	4.558	-3.703***
			(0.432)			(0.555)			(0.435)
Housewife	0.479	0.000	0.479***	0.839	0.012	0.827***	0.707	0.012	0.695***
			(0.060)			(0.041)			(0.052)
Self-Employed	0.085	0.141	-0.057	0.057	0.198	-0.140***	0.122	0.321	-0.199***
			(0.052)			(0.050)			(0.063)
Day Laborer	0.014	0.179	-0.165***	0.103	0.733	-0.629***	0.159	0.631	-0.472***
			(0.046)			(0.058)			(0.067)
Wage Worker	0.000	0.115	-0.115***	0.000	0.035	-0.035*	0.012	0.024	-0.012
			(0.036)			(0.020)			(0.021)
Unemployed	0.732	0.038	0.694***	0.839	0.023	0.816***	0.707	0.024	0.684***
			(0.057)			(0.043)			(0.053)
Student	0.169	0.526	-0.357***	0.000	0.012	-0.012	0.000	0.000	0.000
			(0.072)			(0.012)			(0.000)
N	74	79		87	87		87	87	

Robust standard errors clustered at the individual level in parentheses. * $p < 0.10$, ** $p < 0.05$, *** $p < 0.01$. [†] Marital status here refers to marital status collected at the time of the time use data collection exercise, while column headers refer to baseline marital status from the RCT that collected this information that was used as a demographic strata for our sample.

Table A4: Demographic Balance

	Visit 2 Subsamples				Visit 3 Subsamples						
	(1)	(2)	(3)	(4)	(5)	(6)	(7)	(8)	(9)	(10)	(11)
	Traditional Method Mean	Hybrid Method Mean	P-value: Traditional vs. Hybrid	N	Gold Standard Method Mean	Traditional Method Mean	Hybrid Method Mean	P-Value: Gold Standard vs. Traditional	P-Value: Gold Standard vs. Hybrid	P-Value: Traditional vs. Hybrid	N
Female	0.492	0.498	0.894	499	0.518	0.473	0.500	0.411	0.743	0.621	497
	(0.032)	(0.032)			(0.039)	(0.039)	(0.039)				
Married	0.684	0.663	0.612	499	0.651	0.685	0.681	0.510	0.562	0.936	497
	(0.029)	(0.030)			(0.037)	(0.036)	(0.036)				
Age	31.100	30.671	0.708	499	30.151	31.236	31.283	0.446	0.406	0.974	497
	(0.842)	(0.779)			(0.946)	(1.064)	(0.978)				
Scheduled Caste household	0.445	0.419	0.570	474	0.399	0.456	0.449	0.308	0.372	0.902	472
	(0.032)	(0.032)			(0.039)	(0.040)	(0.040)				
Scheduled Tribe household	0.071	0.042	0.173	474	0.082	0.063	0.026	0.517	0.026**	0.106	472
	(0.017)	(0.013)			(0.022)	(0.019)	(0.013)				
Other Backward Caste household	0.458	0.521	0.169	474	0.481	0.456	0.526	0.653	0.431	0.216	472
	(0.032)	(0.033)			(0.040)	(0.040)	(0.040)				
Num. household members	5.440	5.664	0.258	497	5.409	5.582	5.590	0.452	0.448	0.972	495
	(0.130)	(0.150)			(0.160)	(0.166)	(0.178)				
Num. household members < age 16	1.752	1.791	0.763	499	1.596	1.903	1.795	0.060*	0.186	0.509	497
	(0.089)	(0.095)			(0.106)	(0.123)	(0.107)				
Num. household members < age 7	0.676	0.627	0.555	499	0.524	0.721	0.699	0.051*	0.085*	0.832	497
	(0.061)	(0.058)			(0.068)	(0.074)	(0.075)				
Household Income	6459.389	7086.572	0.377	450	6469.205	6637.586	7249.671	0.845	0.372	0.491	448
	(490.617)	(512.701)			(599.511)	(619.491)	(634.891)				
Years of Education	5.904	6.024	0.758	496	6.521	5.788	5.572	0.128	0.047**	0.653	494
	(0.274)	(0.278)			(0.339)	(0.342)	(0.336)				
Housewife	0.332	0.352	0.634	488	0.348	0.370	0.307	0.674	0.432	0.227	486
	(0.030)	(0.031)			(0.038)	(0.038)	(0.036)				
Self-Employed	0.156	0.156	1.000	488	0.137	0.136	0.196	0.982	0.150	0.143	486
	(0.023)	(0.023)			(0.027)	(0.027)	(0.031)				
Day Laborer	0.320	0.307	0.770	488	0.329	0.290	0.319	0.449	0.845	0.573	486
	(0.030)	(0.030)			(0.037)	(0.036)	(0.037)				
Wage Worker	0.020	0.041	0.191	488	0.012	0.037	0.043	0.155	0.094*	0.787	486
	(0.009)	(0.013)			(0.009)	(0.015)	(0.016)				
Unemployed	0.389	0.389	1.000	488	0.391	0.414	0.362	0.684	0.587	0.341	486
	(0.031)	(0.031)			(0.039)	(0.039)	(0.038)				
Student	0.115	0.107	0.773	488	0.130	0.123	0.080	0.851	0.138	0.194	486
	(0.020)	(0.020)			(0.027)	(0.026)	(0.021)				

Robust standard errors in parentheses. * p≤ 0.10, ** p≤ 0.05, *** p≤ 0.01.

Table A5: Balance on Gold Standard Time for Visit 2 Assignments

	Visit 2 Assignment			
	(1)	(2)	(3)	(4)
	Traditional Method Mean	Hybrid Method Mean	P-value: Traditional vs. Hybrid	N
Wage Work	0.221	0.245	0.922	499
	(0.209)	(0.177)		
Self Employed	0.890	0.887	0.991	499
	(0.234)	(0.238)		
Working Own Field	0.109	0.100	0.968	499
	(0.130)	(0.126)		
HH Chores Outside HH	2.209	2.109	0.675	499
	(0.276)	(0.265)		
HH Chores Inside HH	5.148	4.782	0.068*	499
	(0.322)	(0.298)		
Sleeping	8.919	9.036	0.451	499
	(0.181)	(0.185)		
Leisure	6.314	6.605	0.309	499
	(0.333)	(0.313)		
Active Care	0.190	0.236	0.689	499
	(0.097)	(0.117)		
Passive Care	0.135	0.299	0.326	499
	(0.142)	(0.133)		

Strata dummies included but not shown; robust standard errors in parentheses.
* p≤ 0.10, ** p≤ 0.05, *** p≤ 0.01.

Table A6: Reference Day 1 Comparisons By Demographic Group

	(1) Wage Work	(2) Self Employed	(3) Working Own Field	(4) HH Chores Outside HH	(5) HH Chores Inside HH	(6) Sleeping	(7) Leisure	(8) Active Care	(9) Passive Care	(10) Joint test p-value (χ^2)	(11) Root MSE
Unmarried daughter											
β_1: Traditional Module	-0.128	0.077	0.047	0.135	-0.086	-0.383**	0.189	0.149	0.885*	0.099*	
	(0.230)	(0.353)	(0.209)	(0.334)	(0.341)	(0.153)	(0.320)	(0.098)	(0.466)		
β_2: Hybrid Module	0.500*	-0.529	0.135*	-0.561**	0.131	-0.095	0.412	0.007	-0.084	0.072*	-0.167
	(0.296)	(0.333)	(0.079)	(0.275)	(0.301)	(0.190)	(0.417)	(0.103)	(0.147)		(0.208)
Dependent Var Mean	0.233	1.323	0.105	1.724	4.965	8.977	6.459	0.213	0.217		1.554
N	148	148	148	148	148	148	148	148	148		74
Married woman under 30											
β_1: Traditional Module	0.107	0.163	-0.131	0.246	0.516	0.133	-0.688*	-0.345	1.526**	0.119	
	(0.107)	(0.129)	(0.179)	(0.215)	(0.368)	(0.311)	(0.407)	(0.230)	(0.713)		
β_2: Hybrid Module	-0.098	0.211	-0.052	0.333**	-0.356	-0.344*	0.219	0.087	-1.759**	0.004***	0.023
	(0.128)	(0.182)	(0.041)	(0.165)	(0.268)	(0.199)	(0.371)	(0.193)	(0.789)		(0.261)
Dependent Var Mean	0.200	1.174	0.500	0.763	6.398	8.318	5.148	1.499	3.350		1.932
N	174	174	174	174	174	174	174	174	174		87
Married woman over 30											
β_1: Traditional Module	0.000	0.076	-0.023	-0.055	-0.058	-0.091	-0.336	0.487*	0.178	0.563	
	(0.033)	(0.262)	(0.236)	(0.268)	(0.238)	(0.149)	(0.350)	(0.272)	(0.351)		
β_2: Hybrid Module	-0.262	-0.034	0.091	-0.126	0.554*	-0.159	0.277	-0.341	0.317	0.166	0.194
	(0.163)	(0.305)	(0.191)	(0.325)	(0.322)	(0.244)	(0.367)	(0.445)	(0.297)		(0.212)
Dependent Var Mean	0.500	2.427	0.765	1.317	4.309	8.514	5.588	0.580	0.516		1.473
N	172	172	172	172	172	172	172	172	172		86

Column headers for 1-9 denote variable outcomes, reported in hours. Sample includes Reference Day 1 visits only. All regressions are as specified in equation 1, including individual fixed effects. Standard errors clustered at individual level in parentheses. Column 10 indicates the p-value from an χ^2 test that the coefficients across all categories are jointly equal to zero, evaluated using seemingly unrelated regression on individually demeaned data; standard errors for the joint test are similarly clustered at the individual level. Column 11 reports coefficient for Hybrid method in individual-level regression on root of mean squared difference from visit 1 Gold Standard time, where standard errors are robust. Dependent variable mean in columns 1-9 is for the Gold Standard Day 1 visit; in column 11, the dependent variable statistics report the Traditional method value for the outcome variable. * $p< 0.10$, ** $p< 0.05$, *** $p< 0.01$.

Table A7: Reference Day 1 Comparisons By Demographic Group

	(1) Wage Work	(2) Self Employed	(3) Working Own Field	(4) HH Chores Outside HH	(5) HH Chores Inside HH	(6) Sleeping	(7) Leisure	(8) Active Care	(9) Passive Care	(10) Joint test p-value (χ^2)	(11) Root MSE
Unmarried son											
β_1: Traditional Module	-0.041	0.150	-0.011	-0.026	0.143	-0.002	-0.325	0.111	0.004	0.989	
	(0.296)	(0.256)	(0.171)	(0.217)	(0.297)	(0.161)	(0.332)	(0.140)	(0.019)		
β_2: Hybrid Module	0.504	-0.050	-0.292	-0.802**	-1.633***	-0.162	2.146***	0.290	0.175	0.000***	0.910***
	(0.307)	(0.379)	(0.184)	(0.370)	(0.389)	(0.213)	(0.644)	(0.281)	(0.111)		(0.235)
Dependent Var Mean	1.136	1.620	0.824	2.195	2.152	8.437	7.486	0.150	0.006		1.162
N	158	158	158	158	158	158	158	158	158		79
Man married to woman under 30											
β_1: Traditional Module	0.941**	-0.346	-0.844**	0.400	0.257	-0.069	-0.591**	0.252	0.485**	0.011**	
	(0.446)	(0.395)	(0.396)	(0.323)	(0.170)	(0.239)	(0.265)	(0.157)	(0.225)		
β_2: Hybrid Module	0.526*	-0.141	-0.270	0.012	-0.228	-0.036	0.288	-0.151	0.526*	0.462	0.084
	(0.291)	(0.277)	(0.318)	(0.369)	(0.250)	(0.279)	(0.419)	(0.173)	(0.273)		(0.265)
Dependent Var Mean	2.329	2.544	2.057	1.284	0.609	8.490	6.247	0.440	0.310		1.577
N	174	174	174	174	174	174	174	174	174		87
Man married to woman over 30											
β_1: Traditional Module	-0.100	0.372	0.414	0.305	-0.275*	-0.231	-0.550	0.066	0.244**	0.005***	
	(0.367)	(0.323)	(0.294)	(0.240)	(0.140)	(0.228)	(0.390)	(0.098)	(0.118)		
β_2: Hybrid Module	0.159	0.492	0.147	-0.095	-0.110	0.078	-0.690*	0.019	0.147	0.531	0.044
	(0.198)	(0.335)	(0.456)	(0.335)	(0.121)	(0.367)	(0.387)	(0.045)	(0.113)		(0.220)
Dependent Var Mean	2.070	2.634	2.475	1.159	0.373	8.110	7.058	0.120	0.120		1.522
N	172	172	172	172	172	172	172	172	172		86

Column headers for 1-9 denote variable outcomes, reported in hours. Sample includes Reference Day 1 visits only. All regressions are as specified in equation 1, including individual fixed effects. Standard errors clustered at individual level in parentheses. Column 10 indicates the p-value from an χ^2 test that the coefficients across all categories are jointly equal to zero, evaluated using seemingly unrelated regression on individually demeaned data; standard errors for the joint test are similarly clustered at the individual level. Column 11 reports coefficient for Hybrid method in individual-level regression on root of mean squared difference from visit 1 Gold Standard time, where standard errors are robust. Dependent variable mean in columns 1-9 is for the Gold Standard Day 1 visit; in column 11, the dependent variable statistics report the Traditional method value for the outcome variable. * p< 0.10, ** p< 0.05, *** p< 0.01.

Table A8: Performance with Intrahousehold Controls

	(1) Wage Work	(2) Self Employed	(3) Working Own Field	(4) HH Chores Outside HH	(5) HH Chores Inside HH	(6) Sleeping	(7) Leisure	(8) Active Care	(9) Passive Care	(10) Joint test p-value (χ^2)	(11) Root MSE
γ_1: Traditional Module	0.089	0.018	-0.289	0.402**	-0.160	0.061	-0.326	0.206	0.370*	0.085*	
	(0.160)	(0.174)	(0.185)	(0.185)	(0.182)	(0.147)	(0.239)	(0.128)	(0.218)		
γ_2: Hybrid Module	0.153	-0.056	-0.228	0.045	-0.529***	0.036	0.524*	0.057	-0.305	0.146	0.180*
	(0.186)	(0.212)	(0.158)	(0.195)	(0.192)	(0.167)	(0.292)	(0.185)	(0.259)		(0.097)
γ_3: HH member assigned Traditional method on visit 2	0.043	0.068	0.115	-0.161	0.240**	-0.059	-0.262*	0.016	0.047	0.150	-0.005
	(0.097)	(0.116)	(0.095)	(0.100)	(0.098)	(0.071)	(0.143)	(0.079)	(0.139)		(0.058)
γ_4: HH member assigned Hybrid method on visit 2	0.019	-0.001	0.094	-0.096	0.039	-0.120*	0.168	-0.104	0.148	0.575	0.008
	(0.084)	(0.088)	(0.090)	(0.097)	(0.090)	(0.072)	(0.138)	(0.086)	(0.130)		(0.059)
Gold Standard Mean - Day 1	1.098	1.973	1.150	1.387	3.105	8.463	6.309	0.514	0.781		1.541
Gold Standard SD - Day 1	2.838	2.626	2.691	1.862	3.186	1.730	3.278	1.352	2.214		[1.070]
N	998	998	998	998	998	998	998	998	998		499

Column headers for 1-9 denote variable outcomes, reported in hours. Sample includes all three visits. All regressions are as specified in equation 1 with the addition of the variables noted above. Standard errors clustered at individual level in parentheses. Column 10 indicates the p-value from an χ^2 test that the coefficients across all categories are jointly equal to zero, evaluated using seemingly unrelated regression on individually demeaned data; standard errors for the joint test are similarly clustered at the individual level. Column 11 reports coefficient for Hybrid method in individual-level regression on root of squared difference from visit 1 Gold Standard time, with strata dummies included but not shown. Standard errors in column 11 are robust. * $p < 0.10$, ** $p < 0.05$, *** $p < 0.01$.

Table A9: Heterogeneity by Surveyor and Respondent Gender: (Female Respondents Only)

	(1) Wage Work	(2) Self Employed	(3) Working Own Field	(4) HH Chores Outside HH	(5) HH Chores Inside HH	(6) Sleeping	(7) Leisure	(8) Active Care	(9) Passive Care	(10) Joint test p-value (χ^2)	Root MSE
γ_1: Traditional Module	-0.061	0.149	0.110	-0.138	0.533*	-0.297*	-0.583*	0.288	0.868*	0.029**	
	(0.166)	(0.244)	(0.162)	(0.234)	(0.272)	(0.167)	(0.323)	(0.182)	(0.447)		
γ_2: Traditional Module × Different Gender Surveyor	0.135	-0.104	-0.251	0.480	-0.942**	0.410	0.583	-0.309	-0.079	0.105	
	(0.231)	(0.369)	(0.233)	(0.337)	(0.380)	(0.317)	(0.459)	(0.296)	(0.692)		
γ_3: Hybrid Module	-0.074	0.151	0.227	-0.578**	0.190	-0.159	0.286	-0.042	-0.392	0.174	-0.091
	(0.173)	(0.219)	(0.148)	(0.255)	(0.258)	(0.184)	(0.363)	(0.290)	(0.339)		(0.180)
γ_4: Hybrid Module × Different Gender Surveyor	0.190	-0.467	-0.311	0.918**	-0.193	-0.096	0.006	-0.046	-0.324	0.372	0.229
	(0.182)	(0.344)	(0.210)	(0.368)	(0.425)	(0.272)	(0.525)	(0.307)	(0.741)		(0.264)
γ_5: Different Gender Surveyor	0.020	-0.042	0.353**	-0.250	-0.269	-0.086	-0.202	0.476**	-0.196	0.159	-0.056
	(0.147)	(0.219)	(0.146)	(0.214)	(0.245)	(0.175)	(0.318)	(0.231)	(0.453)		(0.190)
Omitted Group Mean - Same Surveyor Gender	0.303	1.442	0.504	1.276	4.898	8.650	6.127	0.800	1.607		1.679
N	494	494	494	494	494	494	494	494	494		247

Column headers for 1-9 denote variable outcomes, reported in hours. Sample includes Reference Day 1 only. All regressions are as specified in equtaion 1 1, including individual fixed effects and the heterogeneity variable as shown in the table. Standard errors clustered at individual level in parentheses. Column 10 indicates the p-value from a χ^2 test that the coefficients across all categories are jointly equal to zero, evaluated using seemingly unrelated regression on individually demeaned data; standard errors for the joint test are similarly clustered at the individual level. Column 11 reports coefficient for Hybrid method in individual-level regression on square root of sum of squared difference from visit 1 Gold Standard time, with dummies for strata. Standard errors for column 11 are robust. * $p< 0.10$, ** $p< 0.05$, *** $p< 0.01$.

Table A10: Heterogeneity by Surveyor and Respondent Gender: (Male Respondents Only)

	(1) Wage Work	(2) Self Employed	(3) Working Own Field	(4) HH Chores Outside HH	(5) HH Chores Inside HH	(6) Sleeping	(7) Leisure	(8) Active Care	(9) Passive Care	(10) Joint test p-value (χ^2)	Root MSE
γ_1: Traditional Module	0.155	-0.056	0.029	0.255	0.100	-0.055	-0.497**	0.067	0.092	0.611	
	(0.257)	(0.169)	(0.253)	(0.193)	(0.168)	(0.176)	(0.250)	(0.091)	(0.096)		
γ_2: Traditional Module × Different Gender Surveyor	0.409	0.292	-0.518	-0.086	-0.069	-0.105	-0.039	0.117	0.413	0.767	
	(0.571)	(0.536)	(0.470)	(0.321)	(0.270)	(0.296)	(0.495)	(0.218)	(0.275)		
γ_3: Hybrid Module	0.211	0.237	-0.243	-0.192	-0.610***	-0.200	0.780*	0.017	-0.024	0.046**	0.450**
	(0.239)	(0.320)	(0.335)	(0.327)	(0.185)	(0.293)	(0.412)	(0.131)	(0.118)		(0.180)
γ_4: Hybrid Module × Different Gender Surveyor	0.403	-0.273	0.209	-0.197	-0.094	0.343	-0.500	0.109	0.708***	0.154	-0.302
	(0.444)	(0.467)	(0.462)	(0.481)	(0.371)	(0.393)	(0.686)	(0.149)	(0.255)		(0.307)
γ_5: Different Gender Surveyor	-0.046	-0.115	0.247	-0.061	0.277	0.118	-0.103	-0.317*	-0.277*	0.216	0.116
	(0.276)	(0.295)	(0.257)	(0.276)	(0.220)	(0.205)	(0.369)	(0.162)	(0.147)		(0.227)
Omitted Group Mean - Same Surveyor Gender	2.055	2.245	1.883	1.572	0.946	8.247	6.764	0.287	0.131		1.395
N	504	504	504	504	504	504	504	504	504		252

Column headers for 1-9 denote variable outcomes, reported in hours. Sample includes Reference Day 1 only. All regressions are as specified in equtaion 1 1, including individual fixed effects and the heterogeneity variable as shown in the table. Standard errors clustered at individual level in parentheses. Column 10 indicates the p-value from a χ^2 test that the coefficients across all categories are jointly equal to zero, evaluated using seemingly unrelated regression on individually demeaned data; standard errors for the joint test are similarly clustered at the individual level. Column 11 reports coefficient for Hybrid method in individual-level regression on square root of sum of squared difference from visit 1 Gold Standard time, with dummies for strata. Standard errors for column 11 are robust. * p< 0.10, ** p< 0.05, *** p< 0.01.

Figure A1: Mean Squared Error v. Gold Standard Reports by Education Levels

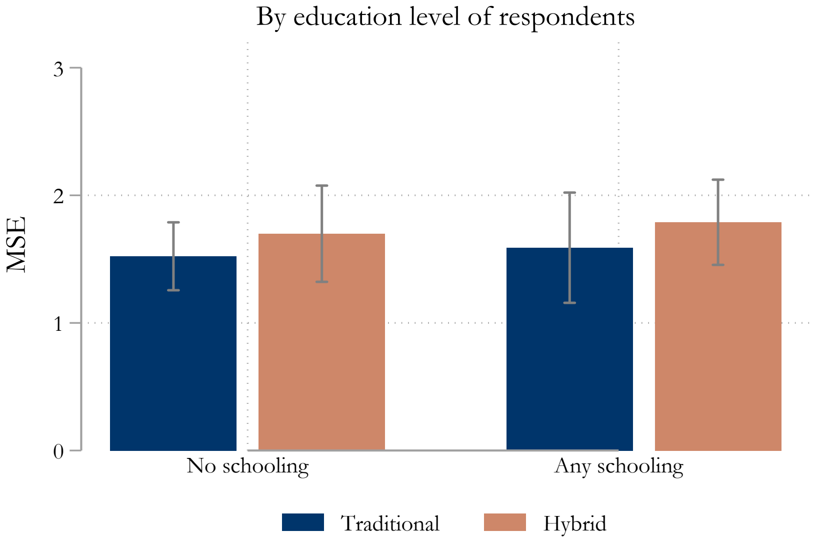

n = 499.
Hybrid, No schooling = Traditional, No schooling: p = 0.114;
Hybrid, Any schooling = Traditional, Any schooling: p = 0.321.
No schooling = Any schooling, Hybrid: p = 0.615.

Graph height shows square root of mean per-person squared error (in hours) from Gold Standard results by method used on visit 2 and listed heterogeneity.
p-values based on robust standard errors.

Figure A2: Cost Estimates for Small Sample Survey

Estimated costs with n = 100 respondents	**Gold Standard**	**Traditional**	**Hybrid**	
Estimated days to complete survey covering 100 respondents	**7**	**5**	**4**	**Comments**
**Training Costs**				
Venue cost	27000	9000	3000	Rs. 3000/day for a training venue; 1 day for Hybrid and 3 days for other methods
Training material cost	225	225	780	3 Rs. Per page; Cost covers pages for survey questionnaire or picture cards x price per print x number of surveyors
Refreshment cost	1500	1500	500	Rs. 100/day*(1, 3 training days)*team of 7
Surveyor salary	8625	8625	2875	Rs.575/surveyor/day x number of training days x 5 surveyors
Supervisor salary	10125	10125	3375	Rs.675 x number of training days x 2 supervisors
**Total Training Cost (INR)**	**₹ 47,475**	**₹ 29,475**	**₹ 10,530**	
**Total Training Cost (USD)**	**$ 742**	**$ 461**	**$ 165**	
**Data Collection Costs**				
**Logistics**				
Car travel	13333	10000	8000	2000 Rs/day rental; full team can fit in the car for a small survey
Surveyor salary	23000	17250	13800	Includes survey for 5 surveyors at 575 Rs./day
Supervisor salary	9000	6750	5400	Survey for 2 supervisors at 675 Rs./day
**Equipment**				
Laptops	444	333	267	Rented for supervisors at 2000 Rs./month
Tablets	2800	2100	1680	Rented for team at 60 Rs./day
SIM cards + recharge	1,000	1,000	1,000	For team survey uploads at end of day, 200 Rs./month
GPS App	1500	0	0	Used to track hourly Gold Standard visits
**Total Data Collection Costs (INR)**	**₹ 51,078**	**₹ 37,433**	**₹ 30,147**	
**Total Data Collection Costs (USD)**	**$ 798**	**$ 585**	**$ 471**	
**Monitoring Costs**				
Motorcycle travel for backchecker	747	560	448	1 motorcycle at Rs.2.8/km, 40Km/day
Backchecker salary	3833	2875	2300	Cost of 575 Rs./day for all backcheck days (survey days - 1), plus 1 training
Tablet	400	300	240	Rented for backchecker
**Total Monitoring & Backchecks Cost (INR)**	**₹ 4,980**	**₹ 3,735**	**₹ 2,988**	
**Total Monitoring & Backchecks Cost (USD)**	**$ 78**	**$ 58**	**$ 47**	
**Total Cost (INR)**	**₹ 103,533**	**₹ 70,643**	**₹ 43,665**	
**Total Cost (USD)**	**$ 1,618**	**$ 1,104**	**$ 682**	

Budget does not include research staff, office costs, overhead, etc. All costs in INR unless otherwise mentioned. 64 INR = 1 USD, in line with the exchange rate at the time of the experiment. We assume productivity of 3 surveys per day for the Gold Standard, 4 for the Traditional method, and 5 for the Hybrid method. Survey team size is assumed to be 5 enumerators, 2 supervisors, and 1 backchecker.

Figure A3: Cost Estimates for Large Sample Survey

Estimated costs with n = 10,000 respondents	**Gold Standard**	**Traditional**	**Hybrid**	
Estimated days to complete survey covering 10,000 respondents	**133**	**100**	**80**	**Comments**
**Training Costs**				
Venue cost	90,000	90,000	30,000	Rs. 15,000/day for a training venue; 1 day for Hybrid and 3 days for other methods
Training material cost	225	225	780	3 Rs. Per page; Cost covers pages for survey questionnaire or picture cards x price per print x number of surveyors
Refreshment cost	15,000	15,000	5,000	Rs. 100/day*(1, 3 training days)*team of 25 surveyors
Surveyor salary	86,250	86,250	28,750	Rs.575/surveyor/day x number of training days x 25 surveyors
Supervisor salary	20,250	20,250	6,750	Rs.675 x number of training days x 5 supervisors
**Total Training Cost (INR)**	**211,725**	**211,725**	**71,280**	
**Total Training Cost (USD)**	**3,160**	**3,160**	**1,064**	
**Data Collection Costs**				
**Logistics**				
Car travel	1,600,000	1,200,000	960,000	2000 Rs/day rental; assume 6 cars needed for 30 person team
Surveyor salary	1,916,667	1,437,500	1,150,000	Payments for 25 surveyors at 575 Rs./day
Supervisor salary	450,000	337,500	270,000	Payments for 5 supervisors at 675 Rs./day
**Equipment**				
Laptops	45,444	34,333	27,000	Rented for supervisors at 2000 Rs./month
Tablets	245,400	185,400	145,800	Rented for team at 60 Rs./day
SIM cards + recharge	30,000	24,000	18,000	For team survey uploads at end of day
GPS App	7,500	-	-	Used to track hourly Gold Standard visits (fixed cost per surveyor)
**Total Data Collection Costs (INR)**	**4,295,011**	**3,218,733**	**2,570,800**	
**Total Data Collection Costs (USD)**	**64,105**	**48,041**	**38,370**	
**Monitoring Costs**				
Motorcycle travel for backcheckers	74,667	56,000	44,800	1 motorcycle at Rs.2.8/km, 40Km/day for 5 backcheckers
Backchecker salary	383,333	287,500	230,000	Cost of 575 Rs./day for all backcheck days (survey days - 1), plus 1 training day
Tablet	40,900	30,900	24,300	Rented for backcheckers at 60 Rs./day
**Total Monitoring & Backchecks Cost (INR)**	**498,900**	**374,400**	**299,100**	
**Total Monitoring & Backchecks Cost (USD)**	**7,446**	**5,588**	**4,464**	
**Total Cost (INR)**	**5,005,636**	**3,804,858**	**2,941,180**	
**Total Cost (USD)**	**74,711**	**56,789**	**43,898**	

Budget does not include research staff, office costs, overhead, etc. All costs in INR unless otherwise mentioned. 64 INR = 1 USD, in line with the exchange rate at the time of the experiment. We assume productivity of 3 surveys per day for the Gold Standard, 4 for the Traditional method, and 5 for the Hybrid method. Survey team size is assumed to be 25 enumerators, 5 supervisors, and 1 backchecker.

A.2. Survey Scripts

Information about Study Visits Given upon Enrollment: If you decide you want to be in this study, we will interview you 3 times over the course of 2 weeks. First, I will come and survey you for an entire day by simply observing what you do in every hour. We will not disturb you in any way or come in the way of your daily activities. We will visit you again on the second day and survey you for about 1 hour. After a few days, we will come again and survey you for about 20-30 minutes.

Enumerator Script, Visit 1: Gold Standard: If you choose to participate in our study then we will visit you at the end of each hour from 8 am till 6 pm and record what activity you engaged in the previous hour. We will only do so for 1-3 days and will ensure that we will only speak to you for 5 minutes and not come in the way while you go about your daily activities. Your participation is extremely valuable to us since the information you provide will help us understand how best to collect information on how people use their time.

Enumerator Script, Traditional Method: We came to you yesterday/a few days ago and observed how you spent your day. Today we wish to interview you about how you spent your day yesterday. Could you please recollect the activities that you took part in since the time you woke up yesterday. I am going to ask you about the activities you did in each hour from 6am yesterday to 6am today morning, and the time spent on multiple activities within each hour. This interview will not last more than one hour.

Enumerator Script, Hybrid Method: Kindly recollect the activities that you took part in since you woke up yesterday. I am going to ask you about the time you spent on broadly these activities - sleeping, income generating activities, household chores (non-income generating activities), child care and leisure.

I have 24 chips representing 24 hours; each chip is equivalent to one hour. I am going to ask you some questions that will help us divide these chips across these activities based on how much time you spent on them.

There is no need to be exact; you can give us approximate time spent on these activities.

1. When did you sleep the day before yesterday and what time did you wake up yesterday? (Surveyor: allocate tokens to the sleep card)

2. What did you do after waking up?

3. How much time did you spend on that activity? (Surveyor: Allocate tokens to the respective picture card)

4. What is the next activity that you did?

For more details on the Hybrid method and protocols, please see the Online Appendix.
