# Supplementary material for: Measuring time use in rural India: Design and validation of a low-cost survey module
Source: J Dev Econ. 2023 Sep;164:103105. doi: 10.1016/j.jdeveco.2023.103105 (PMC10423985; doi:10.1016/j.jdeveco.2023.103105)
Supplement: MMC S2 — . [file mmc2.pdf]

Measuring Time Use in Rural India: Design and Validation of a Low-Cost Survey Module

June 5, 2023

1 Methodological Appendix

1.1 Enumerator Scripts

Introduction to Time Use Data Collection/Entrance into Study

Hello, my name is ... I work for an organization known as J-PAL South Asia based in Gwalior, Madhya Pradesh, which is a research institute at the Institute of Financial Management and Research (IFMR), Chennai. J-PAL is not related to any NGO or government organization.

We are conducting a research study to understand how men and women in Madhya Pradesh use their time. A research study is a way to learn more about something. Through this study, we aim to understand how people, especially those like yourself, usually spend their time throughout the course of a day.

If you decide you want to be in my study, we will interview you 3 times over the course of 2 weeks. First, I will come and survey you for an entire day by simply observing what you do in every hour. We will not disturb you in any way or come in the way of your daily activities. We will visit you again on the second day and survey you for about 1 hour. After a few days, we will come again and survey you for about 20-30 minutes.

We don't think that being in this study will have harm you in any way. Other people will not know if you are in our study. The information I write

down about you and others will be kept safely locked up. When I tell other people or write an article about my research, I will not use your name.

Enumerator Script, Visit 1: Gold Standard: If you choose to participate in our study then we will visit you at the end of each hour from 8 am till 6 pm and record what activity you engaged in the previous hour. We will only do so for 1-3 days and will ensure that we will only speak to you for 5 minutes and not come in the way while you go about your daily activities. Your participation is extremely valuable to us since the information you provide will help us understand how best to collect information on how people use their time.

Enumerator Script, Traditional Method: We came to you yesterday/a few days ago and observed how you spent your day. Today we wish to interview you about how you spent your day yesterday. Could you please recollect the activities that you took part in since the time you woke up yesterday. I am going to ask you about the activities you did in each hour from 6am yesterday to 6am today morning, and the time spent on multiple activities within each hour. This interview will not last more than one hour.

1.2 Enumerator Script, Hybrid Method:

- **The following instructions were given by surveyors to respondents in the field for the Hybrid Method:**

  Kindly recollect the activities that you took part in since you woke up yesterday. I am going to ask you about the time you spent on broadly these activities - sleeping, income generating activities, household chores (non-income generating activities), child care and leisure.

  I have 24 chips representing 24 hours; each chip is equivalent to one hour. I am going to ask you some questions that will help us divide these chips across these activities based on how much time you spent on them.

  There is no need to be exact; you can give us approximate time spent on these activities.

  1. When did you sleep the day before yesterday and what time did you wake up yesterday? (Surveyor: allocate tokens to the sleep card)

2. What did you do after waking up?
3. How much time did you spend on that activity? (Surveyor: Allocate tokens to the respective picture card)
4. What is the next activity that you did?

Apart from this we will also be asking you if you engaged in any passive care of your children. Please note that by passive care we mean you were simply supervising your child but were primarily involved in some other activity. Do not confuse this with active childcare.

For example - if you cooked for 3 hours but, say, for 2 of those hours you also supervised your child who was playing next to you– that would count as passive child care.

- **Surveyors then followed the following protocol for recording passive care time:**

  Surveyor: Kindly fill in the minutes of passive childcare in the relevant activity category or categories. Choose the category where the respondent while engaging in that activity also looked out or supervised her children.

  For example, if while cooking for 2 hours, the woman engaged in passive childcare for 1.5 hours, then in "Household chores inside the house" category record 1.5 x 60 = 90 minutes.

  Please note that passive childcare minutes should not exceed the time you filled in the active activity category in the 24-hour slot.

  For example, if the respondent cooked for 4 hours and engaged in passive care care during that time, that means passive care shouldn't be higher than 4x60=240 minutes.

1.3 Picture Cards and Descriptions used for Hybrid Method

The following images and descriptions were used in the field for the Hybrid method. These images were intended to provide supplementary reference

materials to ensure that respondents of all literacy levels could gain an understanding of the eight activity categories used in the survey.

Time Use Study
FBA Method
Picture Cards: MALE and FEMALE

Pictures showing a man were only used while surveying the men in our sample while pictures showing a woman were only used while surveying the women in our sample. Wherever there is only one picture under a category—that was a common picture used across both male and female surveys.

1. Sleeping
a) At night b) During the day

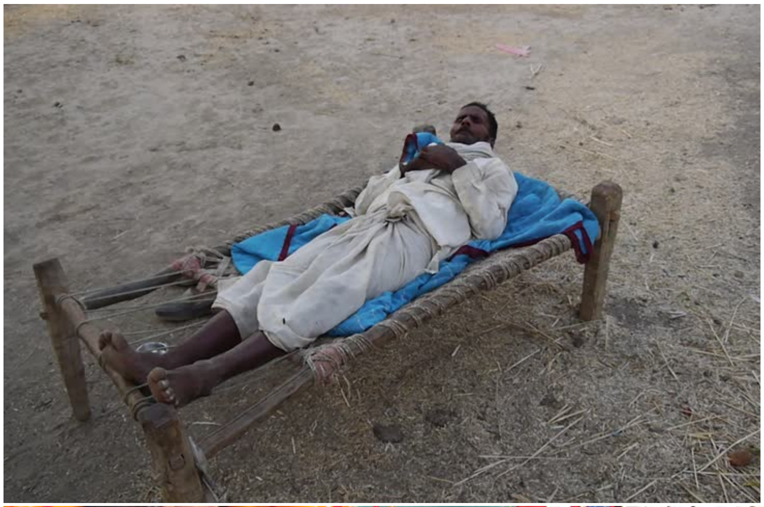

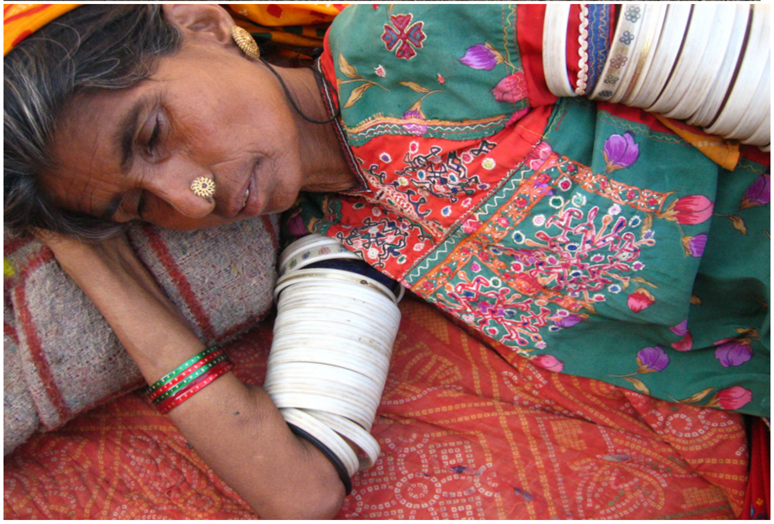

2. Working on own (household's) field

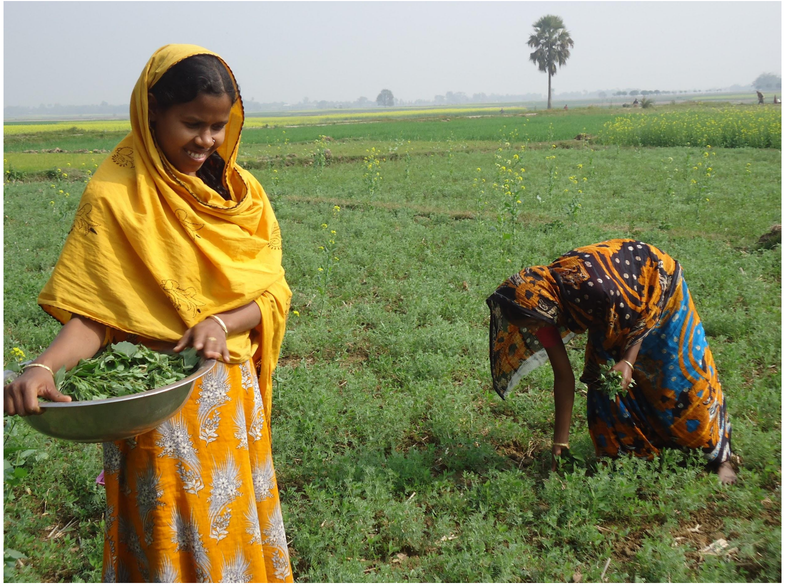

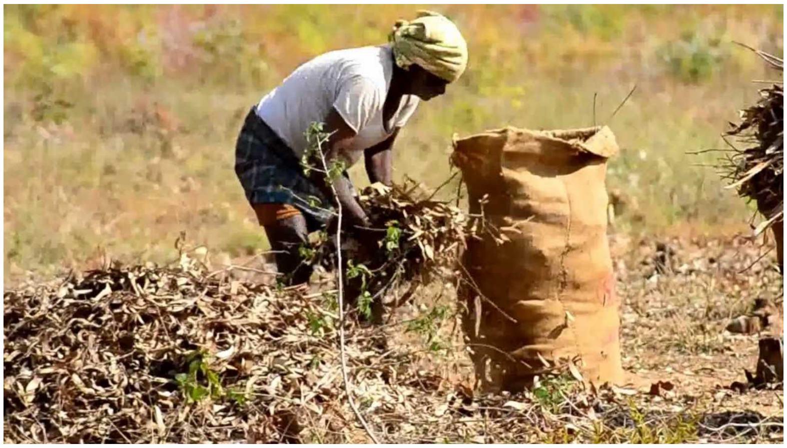

3. Income generating work specific to daily /regular wage

a) Working on someone else's field   b) Construction work c) Other work for pay

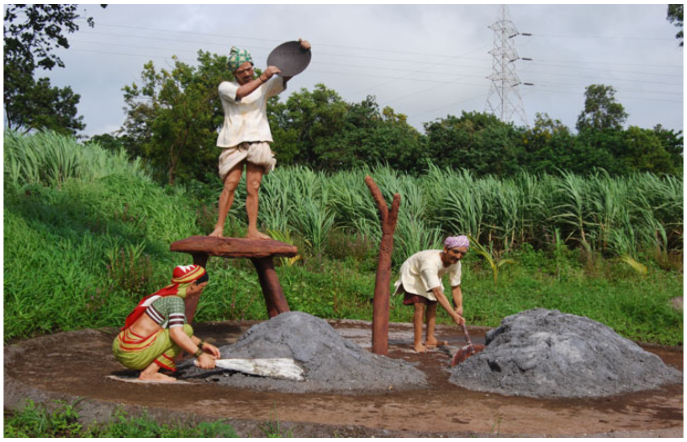

4. Self-employed income generating activities. Like:

a) Cattle rearing b) Making cow dung cakes for selling purpose c) Sewing d) Making baskets e) Making beedis f) Domestic work on someone else's house g) Working as a piece rate worker

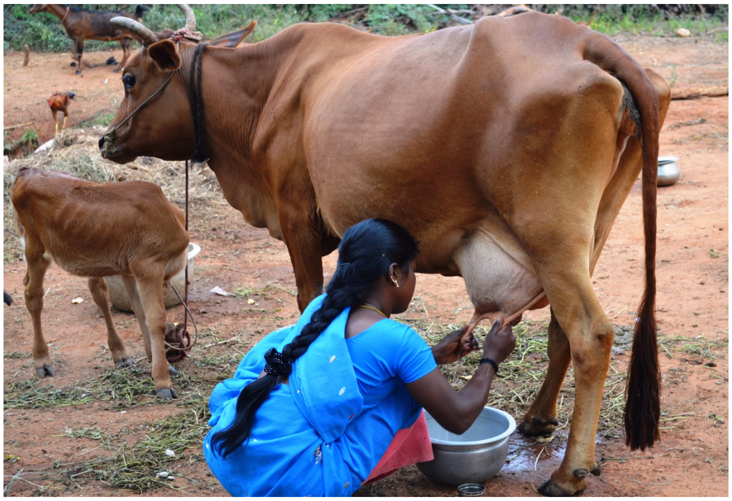

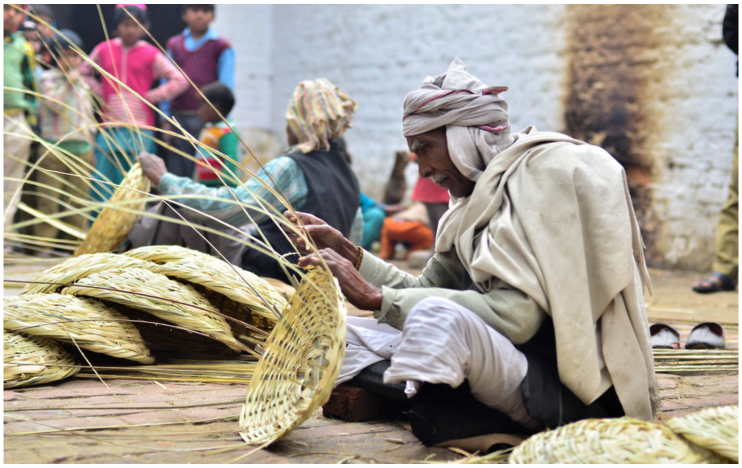

<u>5. Household chores/unpaid work outside the house. Like:</u>

a) Collecting water b) Collecting firewood c) Cleaning clothes at river d) Grocery shopping

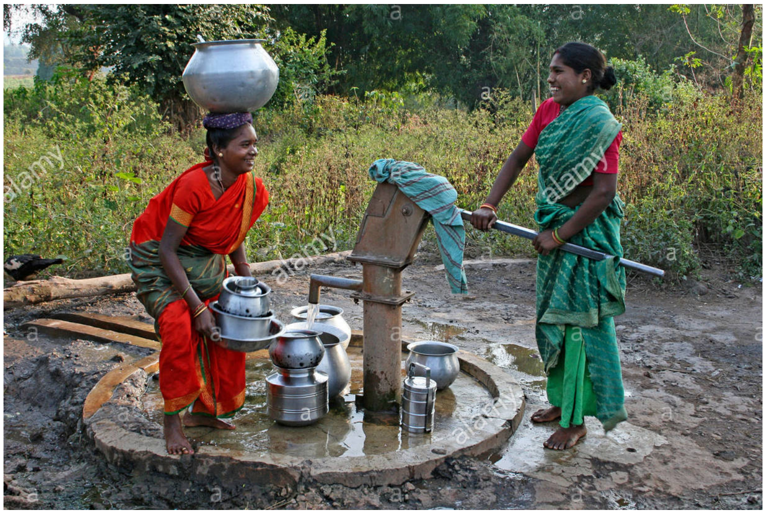

<u>6. Household chores/unpaid work inside the house</u>

Like: a) Cooking b) Cleaning the house (sweeping and mopping) c) Cleaning clothes (inside the house)

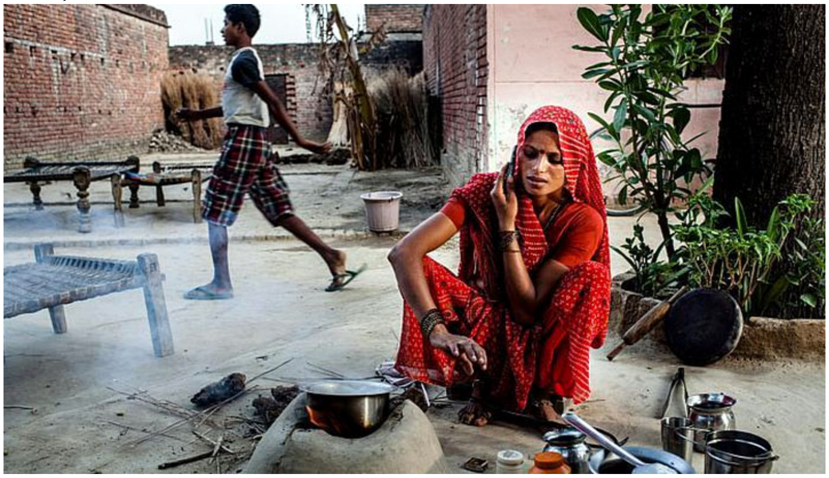

<u>7. Actively taking care/sick/elderly of your children</u>

a) Bathing b) Clothing c) Bringing to school d) Feeding e) Helping with homework f) Playing (actively) with kids (*not* passively playing with the kid while simultaneously working on some other task)

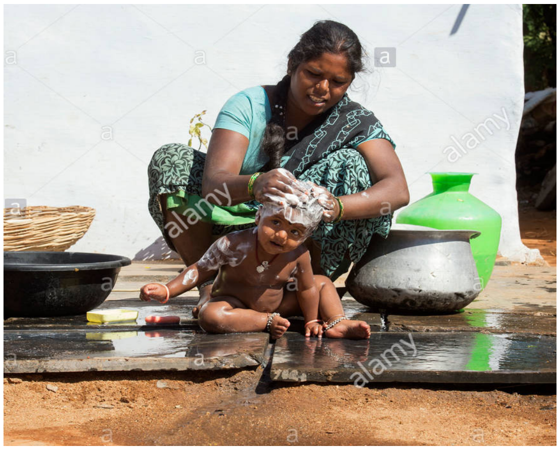

8. Relaxing/Leisure:

a) Resting b) Bathing, getting ready, eating c) Talking to family members or neighbours d) Watching TV or listening to the radio TV e) Attending weddings, funerals and other functions

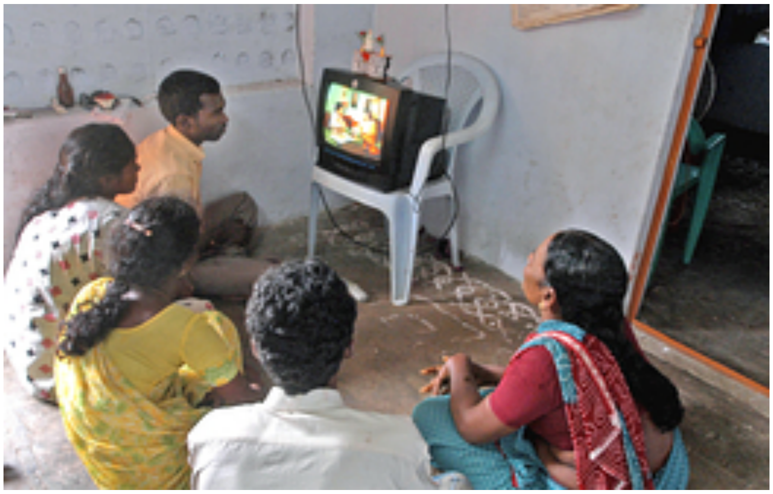

PASSIVE Child Care

Taking care of the child on the side while actively engaged in another activity/task

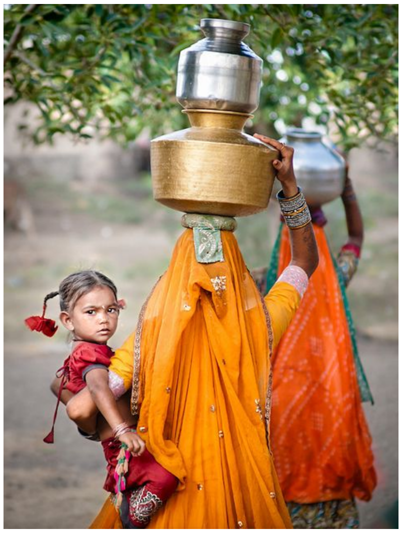

1.4 Activity Crosswalks

Table 1: 8 Hybrid Codes

**Hybrid Code**	**Activity**
1	*Sleeping*<br>a) At night b) Nap during the day
2	*Working on own (household's) field?*
3	*Income generating work specific to daily /regular wage*<br>a) Working on someone elses field b) Construction work c) Other work for pay
4	*Self-employed income generating activities. Like:*<br>a) Cattle rearing b) Making cow dung cakes for selling purpose c) Sewing<br>d) Making baskets e) Making beedis f) Domestic work on someone elses house
5	*Household chores/unpaid work outside the house. Like:*<br>a) Collecting water b) Collecting firewood c) Cleaning clothes at river d) Grocery shopping
6	*Household chores/unpaid work inside the house. Like:*<br>a) Cooking b) Cleaning the house (sweeping and mopping) c) Cleaning clothes (inside the house)
7	*Actively providing care (for children/sick/elderly) with no other activity overlap?*<br>a) Bathing b) Clothing c) Bringing to school<br>d) Feeding e) Helping with homework f) Playing g) Taking care of elders/sick
8	*Relaxing/Leisure:*<br>a) Resting b) Bathing, getting ready, eating c) Talking to family members or neighbours<br>d) Watching TV or listening to the radio TV e) Attending weddings, funerals and other functions

Table 2: Cross-Method Activity Crosswalk -Categories for Traditional Approach, 1998 Indian Time Use Survey

Traditional Code	Activity	Hybrid Code	TUS 98 Hybrid Code
1 [111]	Ploughing, preparing land, cleaning of land	2 if Unpaid<br>3 if Paid	2 if Unpaid<br>3 if Paid
2 [112]	Sewing, planting, transplanting	2 if Unpaid<br>3 if Paid	2 if Unpaid<br>3 if Paid
3 [113]	Application of manure, fertilizer, pesticides and watering, preparing organic/manure. Harvesting, threshing, picking, winnowing	2 if Unpaid<br>3 if Paid	2 if Unpaid<br>3 if Paid
4 [114]	Weeding	2 if Unpaid<br>3 if Paid	2 if Unpaid<br>3 if Paid
5 [115]	Supervision of work	2 if Unpaid<br>3 if Paid	2 if Unpaid<br>3 if Paid
6 [116]	Kitchen gardening - backyard cultivation	2 if Unpaid<br>4 if Paid	2 if Unpaid<br>4 if Paid
7 [117]	Stocking, transporting to home, guarding or protection of crops	2 if Unpaid<br>3 if Paid	2 if Unpaid<br>3 if Paid
8 [118]	Sale and purchase related activities	4	2 if Unpaid<br>3 if Paid
9 [119]	Travel to the work	2 if Unpaid<br>3 if Paid	2 if Unpaid<br>3 if Paid
10 [121]	Grazing animals outside	4	4 if Paid<br>5 if Unpaid
11 [122]	Tending animals - cleaning, washing shed, feeding, watering, preparation of feed.	4	4 if Paid<br>5 if Unpaid
12 [123]	Caring for animals: breeding, shearing, medical treatment, grooming, shoeing etc.	4	4 if Paid<br>5 if Unpaid
13 [124]	Milking and processing of milk. Collecting, storing of poultry products	4	4 if Paid<br>5 if Unpaid
14 [125]	Making dung cakes	4	4 if Paid<br>5 if Unpaid
15 [126]	Poultry rearing - feeding, cleaning.	4	4 if Paid<br>5 if Unpaid
16 [127]	Other related activities.	4	4 if Paid<br>5 if Unpaid
17 [128]	Sale and purchase related activities	4	4 if Paid<br>5 if Unpaid
18 [129]	Travel to the work	4	4 if Paid<br>5 if Unpaid
19 [131]	Nursery - seedlings	2 if Unpaid<br>3 if Paid	2 if Unpaid<br>3 if Paid
20 [132]	Planting, tending, processing of trees.	2 if Unpaid<br>3 if Paid	2 if Unpaid<br>3 if Paid
21 [133]	Collecting, storing & stocking of fruits etc.	2 if Unpaid<br>3 if Paid	2 if Unpaid<br>3 if Paid
22 [134]	Wood cutting, chopping & stocking firewood	5 if Unpaid<br>4 if Paid	5 if Unpaid<br>4 if Paid
23 [135]	Fish farming, feeding fish, catching fish, gathering other aquatic life	5 if Unpaid<br>4 if Paid	5 if Unpaid<br>4 if Paid
24 [136]	Care of house plants, indoor and outdoor garden work	5 if Unpaid<br>3 if Paid	5 if Unpaid<br>3 if Paid

25 [137]	Flower gardening -landscaping, maintenance, cutting, collecting, storing	5 if Unpaid<br>3 if Paid	5 if Unpaid<br>3 if Paid
26 [138]	Sale and purchase related activities	4	5 if Unpaid<br>3 if Paid
27 [139]	Traveling to the work	5 if Unpaid<br>4 if Paid	5 if Unpaid<br>4 if Paid
28 [140]	Fetching of water	5	5
29 [141]	Collection of fruits, vegetables, berries, mushrooms etc. edible goods	5 if Unpaid<br>4 if Paid	5 if Unpaid<br>4 if Paid
30 [142]	Collection of minor forest produce, leaves, bamboo, etc.	5 if Unpaid<br>4 if Paid	5 if Unpaid<br>4 if Paid
31 [143]	Collection of fuelfuel woodtwigs.	5 if Unpaid<br>4 if Paid	5 if Unpaid<br>4 if Paid
32 [144]	Collection of raw material for crafts.	5 if Unpaid<br>4 if Paid	5 if Unpaid<br>4 if Paid
33 [145]	Collection of building materials	5 if Unpaid<br>4 if Paid	5 if Unpaid<br>4 if Paid
34 [146]	Collection of fodder	5 if Unpaid<br>4 if Paid	5 if Unpaid<br>4 if Paid
35 [147]	Sale and purchase related activities	4	5 if Unpaid<br>4 if Paid
36 [148]	Collection of other items	5 if Unpaid<br>4 if Paid	5 if Unpaid<br>4 if Paid
37 [149]	Travel to work.	5 if Unpaid<br>4 if Paid	5 if Unpaid<br>4 if Paid
38 [152]	Milling, husking, pounding	5 if Unpaid<br>3 if Paid	5 if Unpaid<br>3 if Paid
39 [153]	Parboiling	5 if Unpaid<br>4 if Paid	5 if Unpaid<br>4 if Paid
40 [154]	Sorting, Grading	5 if Unpaid<br>4 if Paid	5 if Unpaid<br>4 if Paid
41 [155]	Grinding, crushing	5 if Unpaid<br>4 if Paid	5 if Unpaid<br>4 if Paid
42 [156]	Any other related activity	5 if Unpaid<br>4 if Paid	5 if Unpaid<br>4 if Paid
43 [157]	Sales and purchase related activities	4	5 if Unpaid<br>4 if Paid
44 [159]	Travel for the work	4	4
45 [161]	Mining/extraction of salt, Mining/digging/quarrying of stone, slabs, breaking of stones for construction of	5 if Unpaid<br>3 if Paid	5 if Unpaid<br>3 if Paid
46 [162]	Building road, bridges etc.	5 if Unpaid<br>3 if Paid	5 if Unpaid<br>3 if Paid
47 [163]	Digging out clay, gravel and sand	5 if Unpaid<br>3 if Paid	5 if Unpaid<br>3 if Paid
48 [164]	Digging out minerals	5 if Unpaid<br>3 if Paid	5 if Unpaid<br>3 if Paid

49 [165]	Transporting in vehicles	5 if Unpaid<br>3 if Paid	5 if Unpaid<br>3 if Paid
50 [166]	Storing, stocking	5 if Unpaid<br>3 if Paid	5 if Unpaid<br>3 if Paid
51 [167]	Any other related activity	5 if Unpaid<br>3 if Paid	5 if Unpaid<br>3 if Paid
52 [168]	Sale and purchase related activity	3	5 if Unpaid<br>3 if Paid
53 [169]	Travel for the work	5 if Unpaid<br>3 if Paid	5 if Unpaid<br>3 if Paid
54 [211]	Building & construction of dwelling (laying bricks, plastering, thatching, bamboo work, roofing) and maintenance and repairing of dwelling.	5 if Unpaid<br>3 if Paid	5 if Unpaid<br>3 if Paid
55 [212]	Construction and repair of animal shed, shelter for poultry etc.	5 if Unpaid<br>3 if Paid	5 if Unpaid<br>3 if Paid
56 [213]	Construction of wall. storage facility, fencing etc, irrigation work.	5 if Unpaid<br>3 if Paid	5 if Unpaid<br>3 if Paid
57 [214]	Construction of public workscommon infrastructure - roads, buildings, bridges, etc.	5 if Unpaid<br>3 if Paid	5 if Unpaid<br>3 if Paid
58 [217]	Any other activity related.	5 if Unpaid<br>3 if Paid	5 if Unpaid<br>3 if Paid
59 [218]	Sales and purchase related activity	5 if Unpaid<br>3 if Paid	5 if Unpaid<br>3 if Paid
60 [219]	Travel to the work	5 if Unpaid<br>3 if Paid	3 if Paid<br>5 if Unpaid
61 [221]	Food processing and cooking for sale - making pickles. spices and other products; canning fruits, jams & jellies; baking; beverage preparation, selling readymade food etc.	4	4
62 [222]	Butchering, curing, processing, drying storing etc. of meat, fish etc.	4 if Paid<br>6 if Unpaid	4 if Paid<br>6 if Unpaid
63 [223]	Manufacturing of textiles - spinning, weaving, processing of textiles; knitting, sewing, garment making of cotton, wool and other material.	4 if Paid<br>6 if Unpaid	4 if Paid<br>6 if Unpaid
64 [224]	Making handicrafts, pottery, printing and other crafts made primarily with hands. (wood based leather based crafts, embroidery work etc.)	4 if Paid<br>6 if Unpaid	4 if Paid<br>6 if Unpaid
65 [225]	Fitting, installing, tool setting, tool and machinery - moulding, welding, tool making,	4 if Paid<br>5 if Unpaid	4 if Paid<br>5 if Unpaid
66 [226]	Assembling machines, equipment and other products,	4 if Paid<br>5 if Unpaid	4 if Paid<br>5 if Unpaid
67 [227]	Production related work in large and small factories in different industries - as production workers, maintenance workers paid trainees and apprentices, sales, administration and management activities.	3	3
68 [228]	Sale and purchase related activity	4	4 if Paid<br>5 if Unpaid
69 [229]	Travel for the work	4	4
70 [311]	Buying and selling goods - such as capital goods, intermediate goods, consumer durables, consumer goods in the organised and formal sectors.	4	4
71 [312]	Petty trading, street and door to door vending, hawking, shoe cleaning etc.	4	4
72 [313]	Transporting goods in trucks, tempos and motor vehicles.	4	4

73 [314]	Transporting in hand carts, animal carts, cycle rickshaws etc. or manually	4	4
74 [315]	Transport of passenger by motorized and non-motorised transports	4	4
75 [317]	Any other activity	4	4
76 [319]	Travel to work	4	4
77 [321]	Service in Government and semi government organisations (salaried)	3	3
78 [322]	Service in-private organisations (salaried)	3	3
79 [323]	Petty service: domestic servants, sweepers, washers, pujari, barber, cobbler, mali massaging, prostitution, (wages) watching and guarding	3	3
80 [324]	Professional services: medical and educational services (private tution, non formal teaching etc.), financial services and management and technical consultancy services	3	3
81 [325]	Professional services: computer services, Xerox/photocopying services, beauty parlours, hair cutting saloons etc.	3	3
82 [326]	Technical services: plumbing, electrical and electronic repair and maintenance and other related services	3	3
83 [327]	Others	3	3
84 [329]	Travel to work	3	3
85 [N/A]	Preparing and cooking meals	6	6
N/A [411]	Cleaning food items, beverages, and serving	6	6
86 [421]	Cleaning and upkeep of dwelling and surroundings	6	6
87 [422]	Cleaning of utensils	6	6
88 [431]	Care of textiles: sorting, mending, washing, ironing and ordering clothes and linen	6	6
89 [441]	Shopping for goods and non-personal services: capital goods, household appliances, equipment, food and various household supplies.	6	6
90 [451]	Household Management : planning, supervising, paying bills, etc.	6	6
91 [461]	Do-it-yourself home improvements and maintenance, installation, servicing and repair of personal and household goods	6	6
92 [471]	Pet care	6	6
93 [481]	Travel related to household maintenance, management and shopping	5	5
N/A [491]	Household maintenance, management, and shopping not elsewhere classified	5 if Outside Household<br>6 if Within Household	5 if Outside Household<br>6 if Within Household
94 [511]	Physical care of children: washing, dressing, feeding	7	7
95 [521]	Teaching, training and instruction of own children	7	7
96 [531]	Accompanying children to places: school, sports, lessons, etc./PHC/doctor	7	7
97 [541]	Physical care of the sick, disabled, elderly household members: washing, dressing, feeding, helping.	7	7
98 [551]	Accompanying adults to receive personal care services: such as hairdresser's therapy sessions, temple, religious places etc.	7	7

99 [561]	Supervising children needing care - with or without other activities	7	7
100 [562]	Supervising adults needing care - with or without other activities.	7	7
101 [571]	Travel related to care of children	7	7
102 [572]	Travel related to care of adults and others	7	7
103 [581]	Taking care of guests/visitors	7	7
104 [591]	Any other activity not mentioned above	7	7
105 [611]	Community organised construction and repairs: buildings, roads, dams, wells, ponds etc. community assets.	5 if Unpaid<br>3 if Paid	5 if Unpaid<br>3 if Paid
106 [621]	Community organised work: cooking for collective celebrations, etc.	5 if Unpaid<br>3 if Paid	5 if Unpaid<br>3 if Paid
107 [631]	Volunteering with for an organisation (which does not involve working directly for individuals)	5 if Unpaid<br>3 if Paid	5 if Unpaid<br>3 if Paid
108 [641]	Volunteer work through organisations extended directly to individuals and groups	5 if Unpaid<br>3 if Paid	5 if Unpaid<br>3 if Paid
109 [651]	Participation in meetings of local and informal groups/caste, tribes, professional associations, union fratemal and political organisations	5 if Unpaid<br>3 if Paid	5 if Unpaid<br>3 if Paid
110 [661]	Involvement in civic and related responsibilities: voting, rallies, attending meetings, panchayat	5 if Unpaid<br>3 if Paid	5 if Unpaid<br>3 if Paid
111 [671]	Informal help to other households	5 if Unpaid<br>3 if Paid	5 if Unpaid<br>3 if Paid
112 [681]	Community services not elsewhere classified	5 if Unpaid<br>3 if Paid	5 if Unpaid<br>3 if Paid
113 [691]	Travel related to community services	5 if Unpaid<br>3 if Paid	5 if Unpaid<br>3 if Paid
114 [711]	General Education: School/university/other educational institutions attendance	5 if Outside Household<br>6 if Within Household	5 if Outside Household<br>6 if Within Household
115 [721]	Studies, homework and course review related to general education	5 if Outside Household<br>6 if Within Household	5 if Outside Household<br>6 if Within Household
116 [731]	Additional study, non-formal education under adult education programmes.	5 if Outside Household<br>6 if Within Household	5 if Outside Household<br>6 if Within Household
117 [741]	Non formal education by children	5 if Outside Household<br>6 if Within Household	5 if Outside Household<br>6 if Within Household
118 [751]	Work-related training	5 if Outside Household<br>6 if Within Household	5 if Outside Household<br>6 if Within Household
119 [761]	Training under government programmes such as TRYSEM, DWCRA and others.	5 if Outside Household<br>6 if Within Household	5 if Outside Household<br>6 if Within Household
120 [771]	Other training/education	5 if Outside Household<br>6 if Within Household	5 if Outside Household<br>6 if Within Household
121 [781]	Learning not elsewhere classified	5 if Outside Household<br>6 if Within Household	5 if Outside Household<br>6 if Within Household
122 [791]	Travel related to learning	5 if Outside Household<br>6 if Within Household	5 if Outside Household<br>6 if Within Household

123 [811]	Participating in social events: wedding, funerals, births, and other celebrations	8	8
124 [812]	Participating in religious activities: Church services, religious ceremonies, practices, kirtans, singing, etc.	8	8
125 [813]	Participating in community functions in music, dance etc.	8	8
126 [814]	Socializing at home and outside the home.	8	8
127 [821]	Arts, making music, hobbies and related courses:	8	8
128 [822]	Indoor and outdoor sports participation and related	8	8
N/A [831]	Games and other pastimes	8	8
129 [832]	Spectator to sports, exhibitions/museums, cinema/theatre/concerts and other performances and events	8	8
130 [841]	Other related activities.	8	8
131 [851]	Reading, other than newspaper and magazines.	8	8
132 [852]	Watching television and video	8	8
133 [853]	Listening to music/radio	8	8
134 [861]	Accessing information by computing	8	8
135 [862]	Visiting library	8	8
136 [863]	Reading newspaper, magazines	8	8
137 [871]	Mass media use and entertainment not classified elsewhere such as mobile phone usage, social media	8	8
138 [891]	Travel related to social, cultural and recreational activities, social, cultural and recreational activities, Social, cultural and recreational activities not elsewhere classified, mass media use and entertainment.	8	8
139 [892]	Travel relating to search of jobs.	8	8
140 [911]	Sleep and related activities	1	1
141 [921]	Eating and drinking	8	8
142 [922]	Smoking, drinking alcohol and other intoxicants.	8	8
143 [931]	Personal Hygiene and health	8	8
144 [932]	Walking, exercise mining, jogging, yoga, etc.	8	8
145 [941]	Receiving medical and personal care from professional	8	8
146 [942]	Receiving medical and personal care from household members.	8	8
147 [951]	Talking, gossiping and quarreling	8	8
148 [961]	Doing nothing, rest and relaxation	8	8
149 [971]	Individual religious practices and meditation	8	8
150 [981]	Other activities	8	8

151 [982]	Resting/convalescing due to physical illness and physically unwell persons	8	1
152 [992]	Travel related to personal care and self-maintenance	8	8

Note: 1998 India Time Use Survey activity codes in brackets. Activity codes used in our own data outside brackets. In the case of activities that do not have payment status, we assume the activity is unpaid.

Table 3: Cross-Method Activity Crosswalk -Categories for Traditional Approach, 2019 Indian Time Use Survey

Traditional Code	Activity	Hybrid Code
110	Employment in corporations, government and non-profit institutions	3 if Wage Work<br>4 if Self-employed
121	Growing of crops for the market in household enterprises	3 if Wage Work<br>2 if Self-employed
122	Raising animals for the market in household enterprises	3 if Wage Work<br>4 if Self-employed
123	Forestry and logging for the market in household enterprises	3 if Wage Work<br>4 if Self-employed
124	Fishing for the market in household enterprises	3 if Wage Work<br>4 if Self-employed
125	Aquaculture for the market in household enterprises	3 if Wage Work<br>4 if Self-employed
126	Mining and quarrying for the market in household enterprises	3 if Wage Work<br>4 if Self-employed
127	Making and processing goods for the market in household enterprises	3 if Wage Work<br>4 if Self-employed
128	Construction activities for the market in household enterprises	3 if Wage Work<br>4 if Self-employed
129	Other activities related to employment in household enterprises to produce goods	3 if Wage Work<br>4 if Self-employed
131	Vending and trading of goods in household enterprises	3 if Wage Work<br>4 if Self-employed
132	Providing paid repair, installation, maintenance and disposal in households and household enterprises	3 if Wage Work<br>4 if Self-employed
133	Providing paid business and professional services in households and household enterprises	3 if Wage Work<br>4 if Self-employed
134	Transporting goods and passengers for pay or profit in households and household enterprises	3 if Wage Work<br>4 if Self-employed
135	Providing paid personal care services in households and household enterprises	3 if Wage Work<br>4 if Self-employed
136	Providing paid domestic services	3 if Wage Work<br>4 if Self-employed

139	Other activities related to employment in households and household enterprises providing services	3 if Wage Work<br>4 if Self-employed
141	Activities ancillary to employment	3 if Wage Work<br>4 if Self-employed
142	Breaks during working time within employment	3 if Wage Work<br>4 if Self-employed
150	Training and studies in relation to employment	5 if Outside Household<br>6 if Within Household
160	Seeking employment	8
170	Setting up a business	4
181	Employment-related travel	4
182	Commuting	4
211	Growing crops and kitchen gardening, for own final use	2
212	Farming of animals and production of animal products, for own final use	5
213	Hunting, trapping and production of animal skins, for own final use	5
214	Forestry and logging, for own final use	5
215	Gathering wild products, for own final use	5
216	Fishing, for own final use	5
217	Aquaculture, for own final use	5
218	Mining and quarrying, for own final use	5
221	Making, processing food products, beverages and tobacco for own final use	6
222	Making, processing textiles, wearing apparel, leather and related products, for own final use	6
223	Making, processing of wood and bark products, for own final use	5
224	Making, processing bricks, concrete slabs, hollow blocks, tiles for own final use	5

225	Making, processing herbal and medicinal preparations for own final use	6
226	Making, processing metals and metal products for own final use	5
227	Making, processing of products using other materials for own final use	5
229	Acquiring supplies and disposing of products and other activities related to making and processing goods for own final use	5
230	Construction activities for own final use	5
241	Gathering firewood and other natural products used as fuel for own final use	5
242	Fetching water from natural and other sources for own final use	5
250	Travelling, moving, transporting or accompanying goods or persons related to own-use production of goods	5
311	Preparing meals/snacks	6
312	Serving meals/snacks	6
313	Cleaning up after food preparation/meals/snacks	6
314	Storing, arranging, preserving food stocks	6
319	Other activities related to food and meals management and preparation	6
321	Indoor cleaning	6
322	Outdoor cleaning	5
323	Recycling and disposal of garbage	5
324	Upkeep of in/outdoor plants, hedges, garden, grounds, landscape, etc.	5
325	Tending furnace, boiler, fireplace for heating and water supply	6
329	Other activities related to cleaning and upkeep of dwelling and surroundings	6
331	Do-it-yourself improvement, maintenance and repair of own dwelling	5

332	Installation, servicing and repair of personal and household goods including ICT equipment	5
333	Vehicle maintenance and repairs	5
339	Other activities related to do-it-yourself decoration, maintenance and repair	5
341	Hand/machine-washing	5
342	Drying; hanging out, bringing in wash	5
343	Ironing/pressing/folding	6
344	Mending/repairing and care of clothes and shoes; cleaning and polishing shoes	6
349	Other activities related to care of textiles and footwear	6
351	Paying household bills	6
352	Budgeting, planning, organizing duties and activities in the household	6
359	Other activities related to household management	6
361	Daily pet care	5
362	Using veterinary care or other pet care services (grooming, stabling, holiday or day care)	5
369	Other activities related to pet care	5
371	Shopping for/purchasing of goods and related activities	5
372	Shopping for/availing of services and related activity	5
380	Travelling, moving, transporting or accompanying goods or persons related to unpaid domestic services for household and family members	5
390	Other unpaid domestic services for household and family members	5
411	Caring for children including feeding, cleaning, physical care	7
412	Providing medical care to children	7
413	Instructing, teaching, training, helping children	7

414	Talking with and reading to children	7
415	Playing and sports with children	7
416	Minding children (passive care)	7
417	Meetings and arrangements with schools and child care service providers	7
419	Other activities related to childcare and instruction	7
421	Assisting dependent adults with tasks of daily living	7
422	Assisting dependent adults with medical care	7
423	Assisting dependent adults with forms, administration, accounts	7
424	Affective/emotional support for dependent adults	7
425	Passive care of dependent adult	7
426	Meetings and arrangements with adult care service providers	7
429	Other activities related to care for dependent adults	7
431	Feeding, cleaning, physical care for non-dependent adult household and family members including for temporary illness	7
432	Affective/emotional support for non-dependent adult household and family members	7
439	Other activities related to care for non-dependent adult household and family members	7
441	Travelling related to care-giving services for household and family members	7
442	Accompanying own children	7
443	Accompanying dependent adults	7
444	Accompanying non-dependent adult household and family members	7
490	Other activities related to unpaid caregiving services for household and family members	7
511	Unpaid volunteer household maintenance, management, construction, renovation and repair	5 if Outside Household<br>6 if Within Household

512	Unpaid volunteer shopping/purchasing goods and services	5
513	Unpaid volunteer childcare and instruction	5 if Outside Household<br>6 if Within Household
514	Unpaid volunteer care for adults	5 if Outside Household<br>6 if Within Household
515	Unpaid volunteer unpaid help in enterprises owned by other households	5
519	Other activities related to direct unpaid volunteering for other households	5
521	Unpaid volunteer work on road/building repair, clearing and preparing land, cleaning (streets, markets, etc.), and construction	5
522	Unpaid volunteer preparing/serving meals, cleaning up	5
523	Unpaid volunteer cultural activities, recreation and sports activities	5
524	Unpaid volunteer office/administrative work	5
529	Other activities related to community- and organization-based unpaid volunteering	5
530	Unpaid trainee work and related activities	5
540	Travelling time related to unpaid volunteer, trainee and other unpaid work	5
590	Other unpaid work activities	5
611	School/university attendance	5
612	Extra-curricular activities	5
613	Breaks at place of formal education	5
614	Self-study for distance education course work (video, audio, online)	6
619	Other activities related to formal education	5 if Outside Household<br>6 if Within Household
620	Homework, being tutored, course review, research and activities related to formal education	5 if Outside Household<br>6 if Within Household

630	Additional study, non-formal education and courses	5 if Outside Household<br>6 if Within Household
640	Travelling time related to learning	5
690	Other activities related to learning	5 if Outside Household<br>6 if Within Household
711	Talking, conversing, chatting	8
712	Socializing/getting together/gathering activities	8
713	Reading and writing mail (including email)	8
719	Other activities related to socializing and communication	8
721	Participating in community celebrations of cultural/historic events	8
722	Participating in community rites/events (non-religious) of weddings, funerals, births and similar rites-of-passage	8
723	Participating in community social functions (music, dance, etc.)	8
729	Other activities related to community participation	8
730	Involvement in civic and related responsibilities	8
741	Private prayer, meditation and other spiritual activities	8
742	Participating in collective religious practice	8
749	Other activities related to religious practice	8
750	Travelling time related to socializing and communication, community participation and religious practice	8
790	Other activities related to socializing and communication, community participation and religious practice	8
811	Attendance at organized/mass cultural events, and shows	8
812	Attendance at parks/gardens	8
813	Attendance at sports events	8
819	Other activities related to attendance at cultural, entertainment and sports events	8

821	Visual, literary and performing arts (as hobby)	8
822	Hobbies	8
823	Playing games and other pastime activities	8
829	Other activities related to cultural participation, hobbies, games	8
831	Participating in sports	8
832	Exercising	8
841	Reading for leisure	8
842	Watching/listening to television and video	8
843	Listening to radio and audio devices	8
849	Other activities related to mass media use	8
850	Activities associated with reflecting, resting, relaxing	8
860	Travelling time related to culture, leisure, mass-media and sports practices	8
890	Other activities related to culture, leisure, mass-media and sports practices	8
911	Night sleep/essential sleep	1
912	Incidental sleep/naps	1
913	Sleeplessness	1
919	Other sleep and related activities	1
921	Eating meals/snack	8
922	Drinking other than with meal or snack	8
931	Personal hygiene and care	8
932	Health/medical care to oneself	8
939	Other activities related to personal hygiene and care	8
941	Receiving personal care from others	8

942	Receiving health/medical care from others	8
949	Other activities related to receiving personal and health/medical care	8
950	Travelling time related to self-care and maintenance activities	8
990	Other self-care and maintenance activities	8
